# North Atlantic surface ocean warming and salinization in response to middle Eocene greenhouse warming

**DOI:** 10.1126/sciadv.abq0110

**Published:** 2023-01-25

**Authors:** Robin van der Ploeg, Margot J. Cramwinckel, Ilja J. Kocken, Thomas J. Leutert, Steven M. Bohaty, Chris D. Fokkema, Pincelli M. Hull, A. Nele Meckler, Jack J. Middelburg, Inigo A. Müller, Donald E. Penman, Francien Peterse, Gert-Jan Reichart, Philip F. Sexton, Maximilian Vahlenkamp, David De Vleeschouwer, Paul A. Wilson, Martin Ziegler, Appy Sluijs

**Affiliations:** ^1^ Department of Earth Sciences, Faculty of Geosciences, Utrecht University, Utrecht, Netherlands.; ^2^ Bjerknes Centre for Climate Research and Department of Earth Science, University of Bergen, Bergen, Norway.; ^3^ University of Southampton, Waterfront Campus, National Oceanography Centre, Southampton, UK.; ^4^ Department of Geology and Geophysics, Yale University, New Haven, CT, USA.; ^5^ Department of Geosciences, Utah State University, Logan, UT, USA.; ^6^ NIOZ Royal Netherlands Institute for Sea Research and Utrecht University, Den Burg, Texel, Netherlands.; ^7^ School of Environment, Earth & Ecosystem Sciences, The Open University, Milton Keynes, UK.; ^8^ MARUM – Center for Marine and Environmental Sciences, University of Bremen, Bremen, Germany.; ^9^ Institute of Geology and Paleontology, University of Münster, Münster, Germany.

## Abstract

Quantitative reconstructions of hydrological change during ancient greenhouse warming events provide valuable insight into warmer-than-modern hydrological cycles but are limited by paleoclimate proxy uncertainties. We present sea surface temperature (SST) records and seawater oxygen isotope (δ^18^O_sw_) estimates for the Middle Eocene Climatic Optimum (MECO), using coupled carbonate clumped isotope (Δ_47_) and oxygen isotope (δ^18^O_c_) data of well-preserved planktonic foraminifera from the North Atlantic Newfoundland Drifts. These indicate a transient ~3°C warming across the MECO, with absolute temperatures generally in accordance with trace element (Mg/Ca)–based SSTs but lower than biomarker-based SSTs for the same interval. We find a transient ~0.5‰ shift toward higher δ^18^O_sw_, which implies increased salinity in the North Atlantic subtropical gyre and potentially a poleward expansion of its northern boundary in response to greenhouse warming. These observations provide constraints on dynamic ocean response to warming events, which are consistent with theory and model simulations predicting an enhanced hydrological cycle under global warming.

## Teaser

Integrated paleoclimate reconstructions reveal the dynamic response of the North Atlantic Ocean to ancient greenhouse warming.

## INTRODUCTION

The Eocene epoch (56 to 34 Ma ago) is a potential analog for future climate states (*1*). The early Eocene was characterized by high atmospheric CO_2_ concentrations (*2*), resulting in globally hot climates and reduced meridional temperature gradients (*3*, *4*). This early Eocene greenhouse world was marked by a number of transient warming episodes known as hyperthermal events (*5*), of which the Paleocene-Eocene Thermal Maximum (PETM; ~56 Ma ago) is the most prominent and best studied (*6*–*8*). The Middle Eocene Climatic Optimum (MECO; 40 Ma ago) was the last major warming event of the Eocene, but it remains enigmatic because it was comparatively long-lived (*9*–*12*). Global greenhouse warming is generally thought to result in increased atmospheric moisture content and therefore more vigorous hydrological cycling, as reflected in the “wet-gets-wetter, dry-gets-drier” hypothesis (*13*). However, the regional response to increased radiative forcing may be altered by dynamic changes in atmospheric circulation and is therefore notoriously difficult to capture accurately in paleoclimate model simulations. For example, integrated data–model studies for the Pliocene suggest an important role for weakening of the atmospheric Hadley cell, with wetter instead of drier conditions in the evaporation-dominated subtropical band (*14*). By comparison, substantial evidence exists of increased precipitation at higher latitudes for the early Eocene (*7*, *15*), but patterns are less clear and consistent for the tropics and mid-latitudes (*6*, *16*–*18*). Here, we investigate the response of mid-latitude ocean-atmosphere circulation to MECO warming.

**Fig. 1.**
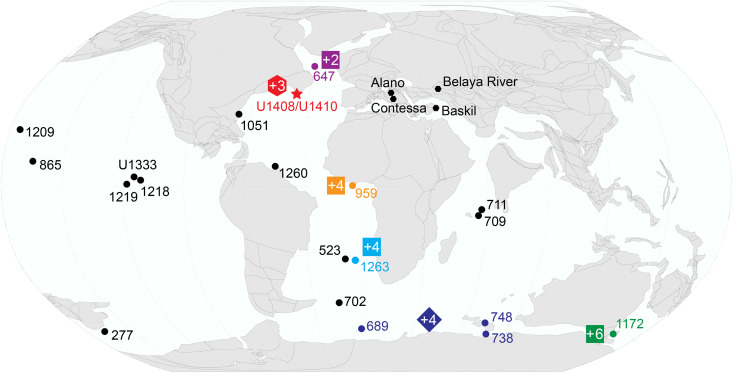
Map of sites with existing MECO records on a paleogeographic reconstruction for the Middle Eocene at 40 Ma. Where available, the magnitude of MECO warming inferred by this study and previous studies (*3*, *9*, *19*, *30*, *31*) is indicated by colored polygons, with +X representing relative warming in degrees Celsius (hexagons based on Δ_47_, squares based on TEX_86_, diamonds based on δ^18^O_c_). The location of Sites U1408 and U1410 is indicated by the red star. Map made with GPlates (*124*) using the paleomagnetic reference frame of (*125*).

Existing proxy records suggest that the MECO greenhouse warming episode included ~400 ka of gradual surface and deep ocean warming ~40 Ma ago, followed by a short peak warming phase and then a comparatively rapid (~100 ka) cooling (*9*–*11*). The warming was associated with a rise in atmospheric partial pressure of CO_2_ (*12*, *19*), carbonate dissolution in the deep ocean (*10*, *11*), and biotic change (*19*–*21*). Both the extended duration of warming and the absence of a negative carbon isotope excursion set the MECO apart from the PETM and other short-lived Eocene hyperthermals (*11*). Reconstructions suggest that the release of CO_2_ into the atmosphere during an episode of enhanced mafic volcanism may have acted as a driver of prolonged MECO warming (*22*). The source of this excess CO_2_ remains elusive, but a recent compilation of radiometric ages of volcanic deposits in Iran and Azerbaijan suggests a link with a continental flare-up in the Neotethys region at that time (*23*). The MECO and/or its associated changes in ocean chemistry and biological communities have now been identified in all major ocean basins (Fig. 1) (*11*). At southern high latitudes, where the event was first described (*9*), 4° to 6°C of warming has been estimated for both the sea surface and the deep sea, on the basis of oxygen isotope ratios of biogenic calcite (δ^18^O_c_) and distributions of alkenones (U^K′^_37_) and isoprenoid glycerol dialkyl glycerol tetraethers (GDGTs; TEX_86_) (*9*, *10*, *19*, *24*). A rise in sea surface temperature (SST) of similar magnitude has subsequently been observed at mid-latitude sites in the former Tethys Ocean (*25*–*28*) and in the North, South, and equatorial Atlantic oceans (*3*, *10*, *29*–*33*). However, with some exceptions, most inferences of MECO SST warming are based on δ^18^O_c_ records of foraminifera or bulk carbonate, which are also affected by (changes in) the oxygen isotope composition of surface ocean seawater (δ^18^O_sw_). Most published records of the MECO are hindered by carbonate dissolution (*10*, *11*), recrystallization (*34*), limited temporal resolution, and/or the relatively poor preservation state of deep-sea sediments (*9*, *10*, *19*, *30*). Moreover, inherent uncertainties persist for individual proxy systems, and it thus remains challenging to accurately reconstruct ocean temperatures and to parameterize and quantify aspects of ocean-atmosphere circulation and the hydrological cycle, all of which are of key importance for testing climate model skill. Deeper insight into these climate dynamics may be achieved by directly reconstructing δ^18^O_sw_, which is sensitive to local water composition (related to salinity) and continental ice-volume effects (if present). Previous studies have extracted δ^18^O_sw_ signals from δ^18^O_c_ records by constraining ocean temperatures via independent proxies (*6*, *16*–*18*), but these attempts also introduce an additional set of proxy-specific uncertainties. Likewise, attempts have been made to simulate steady-state Eocene δ^18^O_sw_ patterns using isotope-enabled climate models (*35*–*37*), but these predictions are, in turn, highly sensitive to boundary conditions such as CO_2_ concentrations and paleogeography and therefore require data for validation.

Here, we present reconstructions of climatic and hydrological change derived from deep-sea sedimentary sequences from Sites U1408 and U1410 from the Newfoundland Drifts in the North Atlantic (see Materials and Methods). These sites are presently located along a strong SST gradient at the northern edge of the North Atlantic subtropical gyre [~41°N; paleolatitude, ~32° ± 3°N; (*38*)], where warm waters from the Gulf Stream meet the much colder waters from the Labrador Sea Current (fig. S1) (*39*). Sediments at the Newfoundland ridges accumulated primarily as drift deposits since ~47 Ma (*40*), and hence, these expanded, clay-rich middle Eocene sediments yield well-preserved foraminifera (fig. S2) and sufficient preservation of organic matter allowing for multiproxy temperature reconstructions. Moreover, because they are located near the present and past (i.e., middle Eocene) boundary between the mid-latitude and adjacent atmospheric circulation cells, these sites are sensitive to effects of climate-induced changes in ocean-atmosphere circulation. The first planktonic foraminiferal stable isotope records for the MECO at Site U1408 were published recently (*32*), but it has now become apparent that a composite record of sections from both Sites U1408 and U1410 is needed to capture the complete MECO interval (fig. S3) (*41*).

We aim to determine the full amplitude and character of sea surface warming and hydrological change during the MECO in the North Atlantic by combining high-resolution clumped isotope (Δ_47_), δ^18^O_c_, and trace element (Mg/Ca) records of planktonic foraminifera with biomarker records based on GDGT distributions (TEX_86_) and alkenones (U^K′^_37_). The δ^18^O_c_, Mg/Ca, U^K′^_37_, and TEX_86_ proxies have been widely used as paleothermometers in Cenozoic paleoclimate studies (*4*), whereas temperature records based on Δ_47_ have only become available in recent years (*42*, *43*). The Δ_47_ proxy is based on the degree of binding between the heavy ^13^C and ^18^O isotopes in carbonate minerals, which is inversely related to temperature (*44*). Unlike δ^18^O_c_ and Mg/Ca, the Δ_47_ composition of carbonates is independent of δ^18^O_sw_ and the elemental composition of seawater, and hence, Δ_47_ has the unique potential of delivering absolute temperature estimates without the need for additional assumptions. Moreover, Δ_47_ compositions of planktonic foraminifera appear unaffected by species-specific vital effects (*45*). Now that consensus is being reached on Δ_47_-temperature calibrations (*46*), the uncertainty in Δ_47_-based temperature reconstructions is controlled mostly by analytical precision, provided that the quality of carbonate preservation is sufficient (*47*) and no postdepositional resetting has occurred. Uniquely for Δ_47_ applications to Eocene paleoclimate reconstructions so far, we report high-resolution planktonic foraminiferal records using large numbers of replicate analyses to reconstruct changes in both SST and δ^18^O_sw_ through time using the exact same analyses.

## RESULTS

### High-resolution foraminiferal clumped isotope records

No single planktonic foraminiferal species is continuously present across the studied MECO interval at both Sites U1408 and U1410 (*32*, *48*, *49*). Therefore, we have constructed a composite record based on three partially overlapping species (groups): *Acarinina (prae-)topilensis*, *Acarinina bullbrooki*, and *Globigerinatheka index*. On the basis of their δ^18^O_c_─δ^13^C_c_ relationships, *A. (prae-)topilensis* and *A. bullbrooki* are both considered to have had photosynthetic algal symbionts and thus reflect surface mixed-layer conditions (fig. S4) (*50*, *51*). *G. index* is also suggested to have symbionts (*32*), but it is likely to have added gametogenic calcite and to have sunk to greater water depths during its reproductive cycle (*52*, *53*), so it may therefore record somewhat lower temperatures from deeper in the ocean (*50*, *53*).

**Fig. 2.**
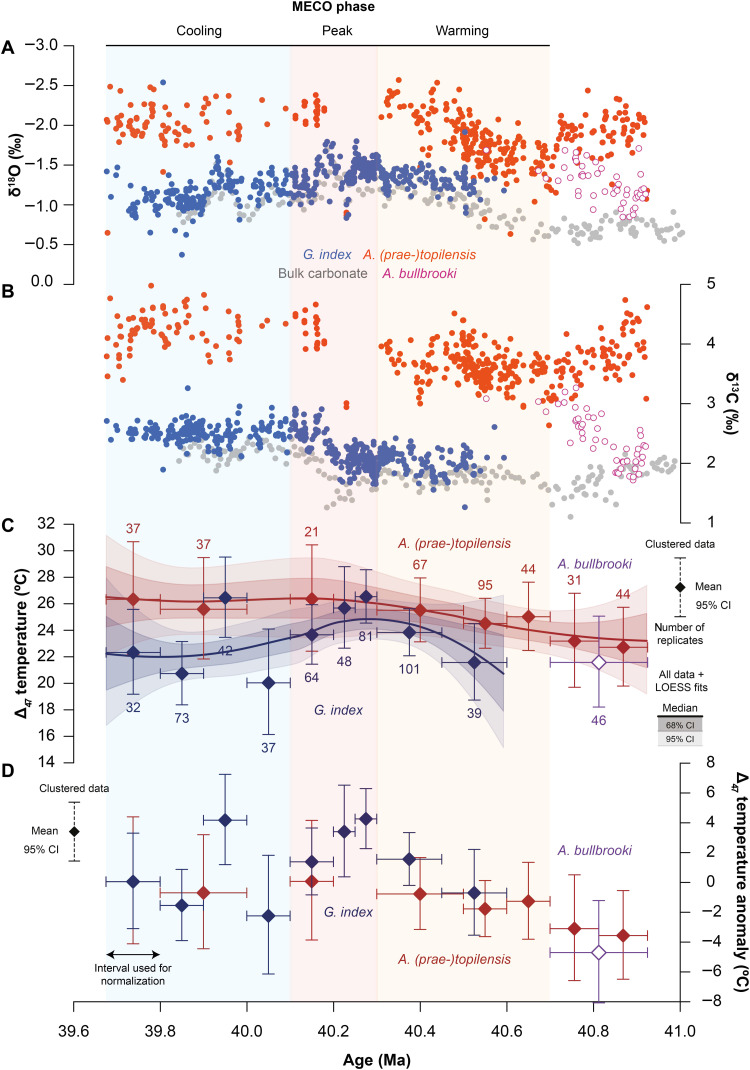
Foraminiferal stable isotope records and clumped isotope-based temperature records for the MECO interval at Sites U1408 and U1410. (**A** and **B**) δ^18^O_c_ and δ^13^C_c_ records for *A. (prae-)topilensis* (in red), *A. bullbrooki* (in purple), and *G. index* (in blue), as well as bulk carbonate (in gray). (**C**) Δ_47_-derived temperatures for *A. (prae-)topilensis* (in dark red), *A. bullbrooki* (in dark purple), and *G. index* (in dark blue). Diamonds represent means of clustered measurements, with vertical error bars indicating 95% confidence intervals (CI), horizontal error bars indicating age ranges of the included measurements, and *n* representing the number of measurements included (see Materials and Methods). Curves represent the median (thick solid lines), 68% CIs (dark shaded areas), and 95% CIs (light shaded areas) of LOESS fits to all measurements for *A. (prae-)topilensis* and *G. index*. (**D**) Δ_47_-derived temperature anomalies for *A. (prae-)topilensis* (in dark red), *A. bullbrooki* (in dark purple), and *G. index* (in dark blue), relative to their respective mean temperatures for the interval between 39.9 and 39.7 Ma. *A. bullbrooki* was normalized to the *A. (prae-)topilensis* value. All records are plotted against age following the revised composite age model for U1408 and U1410.

Across the study interval (~39.6 to 41.0 Ma), our δ^18^O_c_ and δ^13^C_c_ records for these three foraminiferal species groups display trends that are similar to recently published Site U1408 data of lower temporal resolution (fig. S4) (*32*). The combined, multispecies δ^18^O_c_ record shows a total decrease of ~1‰ across the MECO interval (Fig. 2A), which is similar in magnitude to the change observed in the bulk carbonate δ^18^O_c_ record at these sites and most other Atlantic Ocean and Southern Ocean records (*9*, *10*). Notably, on the Newfoundland Margin, this δ^18^O_c_ decrease appears to start as early as 40.7 Ma and is characterized by several distinct minima and maxima rather than the more continuous decrease that was previously observed in other less well-resolved records (*9*, *10*). These variations in δ^18^O_c_ may, in part, be related to orbital forcing recorded in the expanded drift sequences at these sites, but even higher resolution records would be needed to fully resolve this. Cyclic variations in these middle Eocene Newfoundland sediment records have previously been attributed to the obliquity cycle (*54*, *55*), but analyses now show that they most likely reflect the precession cycle instead (*41*). On the basis of the collective δ^18^O_c_ profiles, we assign four phases to the MECO event (background, warming, peak, and cooling, respectively; see Table 1) to facilitate a systematic evaluation of our temperature records in subsequent sections. Notably, on the basis of δ^18^O_c_, the peak MECO warming phase on the Newfoundland Margin appears to occur earlier (i.e., between 40.1 and 40.3 Ma), and the cooling phase appears to be more gradual than in previously published records (*9*, *10*). We observe a gradual increase in δ^13^C_c_ of ~1‰ across the MECO similar in magnitude to other records (Fig. 2B), and during the peak warming interval, we find a subtle δ^13^C_c_ decrease followed by a comparatively rapid δ^13^C_c_ increase in both the *G. index* and bulk carbonate records. This small negative δ^13^C_c_ excursion appears to be coupled with a short-lived increase in δ^18^O_c_. Given the relatively consistent offsets between the δ^18^O_c_ and δ^13^C_c_ values of *A. (prae-)topilensis* and *G. index* across the MECO, we find no evidence for migration of *A. (prae-)topilensis* to greater depths in response to warming (*56*, *57*). Close agreement between the δ^18^O_c_ and δ^13^C_c_ compositions of *G. index* and bulk carbonate further suggests that both records carry similar imprints of environmental change in the studied interval, thus limiting the likelihood that *G. index* would instead record transient changes in gametogenic calcification and/or associated depth shifts. The detailed character and meaning of δ^13^C_c_ change across the MECO are further discussed in other studies (*10*–*12*, *29*, *30*, *32*).

**Table 1.** Comparison of Δ_47_-derived temperatures for *A. (prae-)topilensis*, *A. bullbrooki*, and *G. index* across the MECO warming, peak, and cooling phases. Temperatures are calculated as averages using the age definitions below and using the mean, minimum, and maximum temperatures (95% CI) for each cluster as reported in the Supplementary Materials.

MECO phase	Age (Ma)	Δ_47_-based temperatures			Δ_47_-based temperatures			Δ_47_-based temperatures		
		*A. (prae-)topilensis*			*A. bullbrooki*			*G. index*		
		Mean (°C)	Min (°C)	Max (°C)	Mean (°C)	Min (°C)	Max (°C)	Mean (°C)	Min (°C)	Max (°C)
Cooling	<40.1	26.0	22.0	30.1	–	–	–	22.4	19.3	25.6
Peak	40.1–40.3	26.4	22.9	30.1	–	–	–	25.3	22.9	27.8
Warming	40.3–40.7	25.0	22.8	27.4	–	–	–	22.7	20.4	25.1
Background	>40.7	23.0	19.8	26.3	21.6	18.2	25.1	–	–	–

We present our Δ_47_-based temperature estimates in two ways (see Materials and Methods): as temperature clusters based on Δ_47_ averages of neighboring measurements per foraminiferal species and as smoothed, continuous records based on locally weighted smoothing (LOESS) fits to all available Δ_47_ measurements per foraminiferal species (Fig. 2C, Table 1, and fig. S5). For *A. bullbrooki*, which is only abundantly present in the oldest part of the record, we obtain Δ_47_-based temperatures of 22° ± 3°C [95% confidence interval (CI)] between 40.7 and 40.9 Ma. We obtain very similar Δ_47_-based temperatures for *A. (prae-)topilensis* (i.e., 23° ± 3°C) in this part of the record (40.7 to 40.9 Ma), despite a clear offset of ~1‰ in δ^18^O_c_ values between the two taxa (Fig. 2A and fig. S4). This similarity in reconstructed temperatures might indicate that ecological differences between *A. (prae-)topilensis* and *A. bullbrooki* are smaller than previously inferred from stable isotopic compositions (*50*, *51*), but the limited data for *A. bullbrooki* hinder any further interpretations. We further reconstruct *A. (prae-)topilensis*–derived temperatures of 25° ± 2°C across the MECO warming interval between 40.3 and 40.7 Ma, and temperatures of 26° ± 4°C in the post-MECO interval between 39.7 and 40.1 Ma. We observe the highest absolute temperatures (~26.5° ± 4°C) between 40.1 and 40.2 Ma, but the near absence of *A. (prae-)topilensis* between 40.0 and 40.3 Ma precludes reliable temperature reconstructions in the peak warming phase of the MECO based on this species group. This may either be related to seafloor carbonate dissolution (*58*) during peak MECO warmth, as *A. (prae-)topilensis* has thinner walls compared to other species and carbonate content is generally low in this interval (*48*, *49*), or be related to susceptibility of *A. (prae-)topilensis* to symbiont bleaching (*59*) or surface ocean acidification (*12*). Another explanation for the absence of *A. (prae-)topilensis* may be a thermal range shift related to MECO warming (*29*). However, *G. index* is present through this interval and captures the extent of MECO warming, yielding temperatures of 23° ± 3°C between 40.3 and 40.6 Ma, 25° ± 3°C between 40.1 and 40.3 Ma, and 22° ± 3°C between 39.7 and 40.1 Ma. These *G. index*–derived temperatures are mostly cooler than the *A. (prae-)topilensis*–derived temperatures, which is consistent with the 0.5 to 1.0‰ offset in δ^18^O_c_ between these two species groups, except for a single data point between 39.9 and 40.0 Ma that appears to coincide with a small δ^18^O_c_ decrease during the cooling phase. Because *A. (prae-)topilensis* is mostly absent between 40.0 and 40.3 Ma, the warmest available *A. (prae-)topilensis* temperatures most likely underestimate true peak MECO warmth. Using a conservative approach, we estimate a total upper ocean mixed-layer warming of 3° ± 3°C across the event, although the more complete *G. index* record suggests that a larger magnitude of warming is likely.

### Surface ocean seawater δ^18^O and salinity reconstructions

**Fig. 3.**
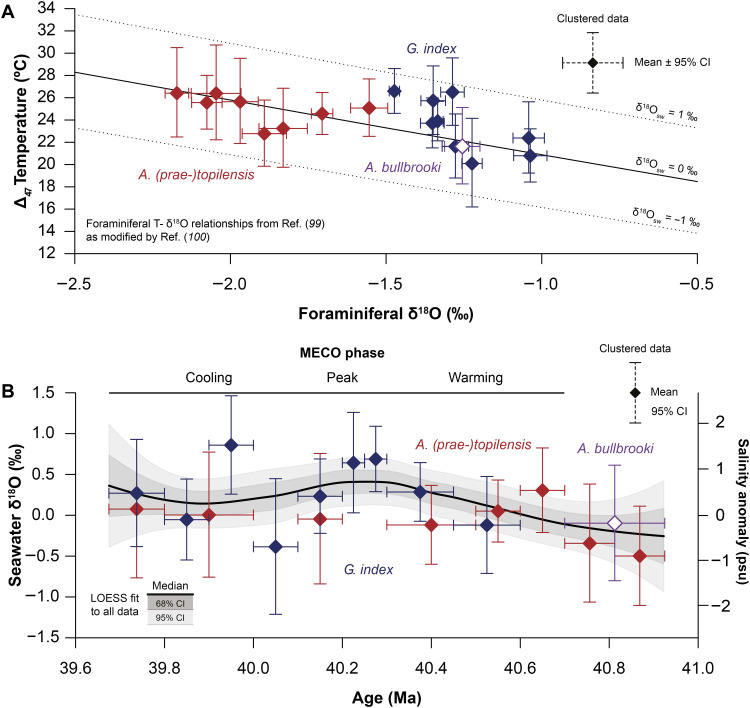
Foraminiferal δ^18^O versus Δ_47_-based temperature relationships and seawater δ^18^O reconstructions for the MECO interval at Sites U1408 and U1410. (**A**) Clustered foraminiferal δ^18^O_c_ values and Δ_47_-based temperatures for *A. (prae-)topilensis* (in dark red), *A. bullbrooki* (in dark purple), and *G. index* (in dark blue), with both vertical and horizontal error bars indicating 95% CIs. Clusters are identical to those in Fig. 2C. Also shown is the foraminiferal δ^18^O_c_-based temperature calibration of (*99*) as modified by (*100*) for δ^18^O_sw_ values of −1, 0, and 1‰ [in Vienna standard mean ocean water (VSMOW)]. (**B**) δ^18^O_sw_ reconstructions based on δ^18^O_c_ values and Δ_47_-derived temperatures for *A. (prae-)topilensis* (in red) and *G. index* (in blue), using the δ^18^O_c_-based temperature calibration of (*99*) as modified by (*100*). Diamonds represent δ^18^O_sw_ estimates from clustered foraminiferal δ^18^O_c_ values and Δ_47_-based temperatures as in (A). Curves represent the median (thick solid lines), 68% CIs (dark shaded areas), and 95% CIs (light shaded areas) of LOESS fits to δ^18^O_sw_ estimates based on individual δ^18^O_c_ values and Δ_47_-based temperatures from all species groups combined. Sea surface salinity reconstructions based on δ^18^O_sw_ estimates and the modern δ^18^O_sw_-salinity relationship of the Atlantic Ocean (*101*), presented as anomalies relative to δ^18^O_sw_ = 0‰.

We use our Δ_47_-based temperature records to reconstruct hydrographic change in the North Atlantic by extracting δ^18^O_sw_ from our foraminiferal δ^18^O_c_ records (Fig. 3A and see Materials and Methods). Crucially, this is the most direct method to estimate δ^18^O_sw_ values currently available for paleoclimate reconstructions. We observe a generally good match between the δ^18^O_sw_ values obtained from *A. (prae-)topilensis* and *G. index*, with considerable changes in δ^18^O_sw_ across the studied interval (Fig. 3B). Because some of the observed variability between δ^18^O_sw_ clusters could be related to analytical uncertainties in both Δ_47_ and δ^18^O_c_, we prefer to use a LOESS fit based on the δ^18^O_sw_ data from all three foraminiferal species groups rather than directly comparing estimates from adjacent clusters. Such a LOESS fit produces a smoothed trend with a transient δ^18^O_sw_ increase of 0.5 ± 0.5‰ across the MECO. By comparison, the individual clusters show a pre-MECO δ^18^O_sw_ value of −0.5 ± 1‰ based on *A. (prae-)topilensis* between 40.7 and 40.9 Ma, a peak MECO warmth δ^18^O_sw_ value of 0.5 ± 1‰ based on *G. index* around ~40.2 Ma, and a post-MECO return to a δ^18^O_sw_ value of 0.0 ± 1‰ based on both *A. (prae-)topilensis* and *G. index* between 39.7 and 39.9 Ma. We note that the single δ^18^O_sw_ data point for *A. bullbrooki* between 40.7 and 40.9 Ma is offset from the adjacent *A. (prae-)topilensis* δ^18^O_sw_ data points. This arises because these species have similar Δ_47_-based temperatures but different δ^18^O_c_ compositions. We further observe that the highest δ^18^O_sw_ data points for *A. (prae-)topilensis* and *G. index* occur between 40.6 and 40.7 Ma and 39.9 and 40.0 Ma, respectively, suggesting that additional short-term δ^18^O_sw_ variability may be present within the record. We also use our δ^18^O_sw_ records to estimate changes in mixed-layer salinity based on present-day δ^18^O_sw_-salinity relationships for the Atlantic Ocean (using a slope of 0.558‰ / practical salinity unit (psu); see Materials and Methods) and conservatively reconstruct a transient sea surface salinity increase of ~1 psu during MECO warming (Fig. 3B).

### Multiproxy SST comparison

**Fig. 4.**
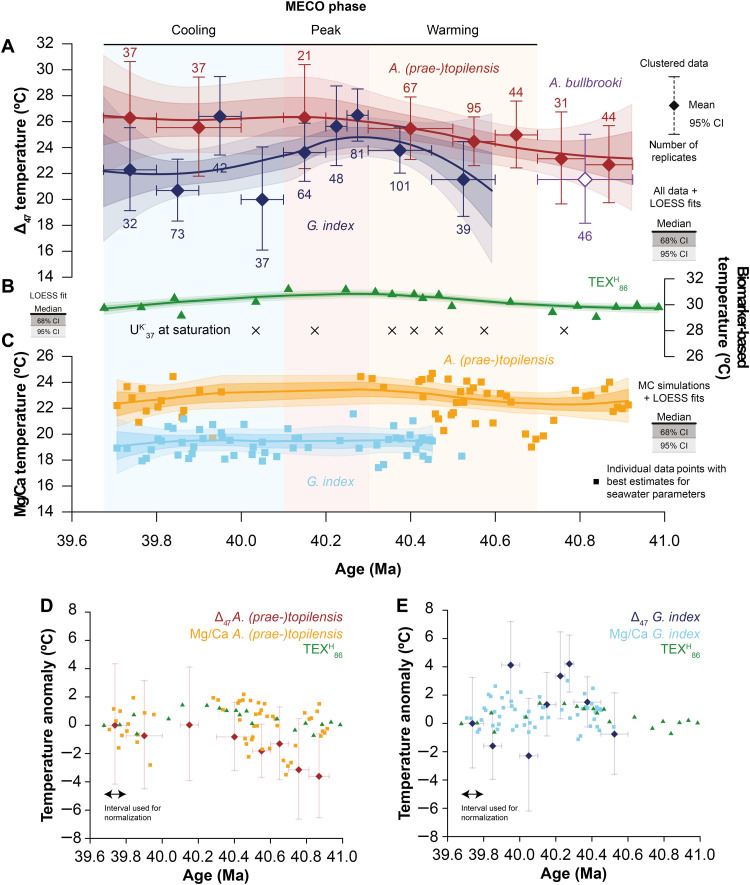
Multiproxy temperature reconstructions for the MECO interval at Sites U1408 and U1410. (**A**) Δ_47_-derived temperatures as presented in Fig. 2C. (**B**) TEX_86_^H^-derived temperatures (in green) and U^K′^_37_-derived temperatures (in black), with the solid green line representing a LOESS fit. Calibration uncertainties for temperatures based on TEX_86_^H^ are ~2.5°C (63). U^K′^_37_ was at proxy saturation in all samples where alkenones could be detected, so the resulting temperatures represent absolute minimum estimates upward of 28°C. (**C**) Mg/Ca-derived temperatures for *A. (prae-)topilensis* (in orange) and *G. index* (in light blue) based on the Mg/Ca-temperature relationships of (*60*), with modifications following (*61*). Squares represent estimates from individual samples based on most probable middle Eocene values for the salinity, pH, and Mg/Ca composition of seawater (salinity = 34 psu, pH = 7.8, and Mg/Ca_sw_ = 2.25 mmol/mol; see Materials and Methods for further details). Curves represent the median (thick solid lines), 68% CIs (dark shaded areas), and 95% CIs (light shaded areas) of LOESS fits to Monte Carlo simulations of Mg/Ca-derived temperatures (*n* = 1000) in which the analytical uncertainties and uncertainties regarding salinity, pH, and Mg/Ca_sw_ are fully propagated (see Materials and Methods). Note that the combined temperature scale of (B) and (C) is equal to the temperature scale of (A). (**D**) Comparison of Δ_47_-derived and Mg/Ca-derived temperature anomalies for *A. (prae-)topilensis* (in dark red and orange, respectively) with TEX_86_^H^-derived temperature anomalies (in green), normalized to their respective mean temperatures for the interval between 39.9 and 39.7 Ma. (**E**) Comparison of Δ_47_-derived and Mg/Ca-derived temperature anomalies for *G. index* (in dark blue and light blue, respectively) with TEX_86_^H^-derived temperature anomalies (in green). Normalization interval as in (D).

In Fig. 4, we compare our Δ_47_-based SST records to our generated SST reconstructions derived from Mg/Ca, TEX_86_, and U^K′^_37_. Our Mg/Ca-based SST estimates are based on *A. (prae-)topilensis* and *G. index* using Mg/Ca-temperature relationships that account for the effects of changes in salinity, pH, and the Mg/Ca composition of seawater (Mg/Ca_sw_) relative to their modern values (see Materials and Methods, Fig. 4C, and figs. S6 and S7) (*60*, *61*). We use Monte Carlo simulations to propagate the associated analytical uncertainties and a range of probable middle Eocene salinity, pH, and Mg/Ca_sw_ values and test for the impact of using different Mg/Ca-temperature calibrations. The resulting Mg/Ca-based records yield similar overall temperatures to the Δ_47_-based records but with a relatively subdued warming across the MECO. For *A. (prae-)topilensis*, we obtain temperatures of ~22° ± 2°C at 40.8 Ma and ~23° ± 2°C at 40.3 Ma, which would suggest a total upper ocean mixed-layer warming of ~1°C. Again, no data are available for the interval between 40.0 and 40.3 Ma owing to the absence of *A. (prae-)topilensis*. For *G. index*, however, we find no significant signs of warming and instead, we observe stable temperatures at 19° ± 2°C across the entire studied interval (40.5 to 39.8 Ma).

We also perform a rigorous evaluation of the branched and isoprenoid GDGT (isoGDGT) distributions in the studied interval (see Materials and Methods) and exclude approximately half of our TEX_86_ dataset based on aberrant values of the Ring Index and other key isoGDGT ratios indicating nonthermal effects on the isoGDGT composition (figs. S8 to S10). Branched and isoprenoid tetraether (BIT) index values of >0.3 throughout the studied interval (fig. S8) suggest a possible contribution of isoGDGTs from land (*62*), although we find no distinct relationship between the BIT index and TEX_86_ in the residual dataset, providing more confidence in the temperature signal. For the observed range of TEX_86_ values (0.7 to 0.8), the exponential TEX_86_^H^ calibration (*63*) and the linear Bayesian SPAtially-varying Regression (BAYSPAR) calibration (*64*) result in very similar absolute temperature estimates. We reconstruct pre-MECO temperatures of 30° ± 2.5°C at 40.8 Ma and peak MECO temperatures of ~31° ± 2.5°C at 40.0 Ma (Fig. 4B), which suggests a very modest sea surface warming of ~1.5°C (~2°C with a linear calibration; see fig. S8). Moreover, for the samples where alkenones could be detected, we obtain U^K′^_37_ values that are consistently at saturation, implying SSTs of at least 28°C (Fig. 4B and see Materials and Methods) (*65*). Similarly high U^K′^_37_ values in this part of the North Atlantic around 40 Ma have been reported from the nearby Site U1404 (*66*). Together, these lines of evidence suggest that the high TEX_86_ and U^K′^_37_ values may be internally consistent, but because of the high brGDGT contributions, both absolute and relative temperature estimates from TEX_86_ should be interpreted with caution.

## DISCUSSION

The pre-MECO δ^18^O_sw_ value for the Newfoundland margin of −0.5‰ in our records is fairly similar to paleo-estimates for the North Atlantic obtained by using present-day surface ocean δ^18^O_sw_ with corrections for middle Eocene latitude and present-day ice-volume (*67*). A δ^18^O_sw_ value of −0.5‰ is also in agreement with the predictions of general circulation model simulations of the Eocene (*35*, *37*). However, the ~0.5‰ shift to higher δ^18^O_sw_ during the MECO implies that assumptions of constant δ^18^O_sw_ under ice-free conditions—as often applied in δ^18^O_c_-based reconstructions of absolute temperatures across Eocene warming events (*68*)—are unjustified, at least for this oceanographic setting (*6*). Assuming no net transfer of water from the ocean to the cryosphere (ice sheets) or terrestrial realm (groundwater) and, thus, a constant global ocean mean δ^18^O_sw_ composition of −1.0‰ across our study interval, a transient increase in δ^18^O_sw_ in the mid-latitude North Atlantic also implies that, elsewhere (e.g., at high latitudes), δ^18^O_sw_ must have decreased, consistent with hydrogen isotope (δD) reconstructions of plant wax biomarkers across early Eocene hyperthermals (*7*, *69*) or with changing oceanographic influences at the sites. These observations are key for the interpretation of δ^18^O_c_ records of the MECO at other sites, which may misrepresent the magnitude of warming if changes in local δ^18^O_sw_ are not considered. In addition, because foraminiferal δ^18^O_c_ compositions are affected by seawater pH (*70*), part of the MECO warming reconstructed from δ^18^O_c_ may be masked by a pH decrease across the event (*12*). We note that such a transient pH decrease could also affect our estimated δ^18^O_sw_ shift: a pH decrease from ~7.8 to ~7.6, as suggested by foraminiferal boron isotope (δ^11^B) records across the MECO (*12*), could potentially result in an overestimation of the δ^18^O_sw_ shift by up to 0.3‰ through the impact of seawater carbonate chemistry on δ^18^O_c_ (*70*). However, this is not sufficient to account for the total δ^18^O_sw_ variability and the magnitude of δ^18^O_sw_ change that we observe in our records.

The shift toward higher surface ocean δ^18^O_sw_ and salinity during MECO warming could be explained by a northward expansion of the saline surface waters characteristic of the North Atlantic subtropical gyre (*35*, *71*). The large SST and salinity gradients observed at the Newfoundland Drifts in the present-day (fig. S1) suggest that slight shifts in the northern front of this subtropical gyre could have a relatively large effect under local oceanographic conditions. This apparent salinization of the mid-latitude North Atlantic, if representative at scale, would be consistent with enhanced atmospheric moisture transport from intensified hydrological cycling, in line with the predictions of salty-gets-saltier and fresh-gets-fresher for warmer-than-modern greenhouse climates (*72*). Similar patterns of warming and salinization have been inferred at low-to-mid latitude sites for the PETM and Eocene Thermal Maximum 2 (ETM-2) hyperthermals (*6*, *18*, *73*). Given the reconstructed paleolatitude of the study sites (~32° ± 3°N) (*38*), increasing δ^18^O_sw_ during the MECO may also indicate poleward expansion of the atmospheric Hadley cell. This tropical circulation cell, which extends to ~30°N in the present day, could have brought warmer and drier conditions to the North Atlantic if its limits moved poleward. However, on regional scales, the relationship between δ^18^O_sw_ and the hydrological cycle can also be affected by changes in ocean circulation and high-latitude ventilation (*37*) and changes in meridional and zonal temperature gradients (*14*). Moreover, as our observations remain limited to one location, we cannot rule out regional oceanographic changes as a driver of the recorded shifts in δ^18^O_sw_ and salinity rather than globally intensified hydrological cycling. Future studies should therefore focus on reconstructing variations in SSTs and the isotopic composition of both seawater and precipitation (δ^18^O and δD) (*74*) in different locations and at a sufficient temporal resolution to further test these relationships.

In addition to reconstructing δ^18^O_sw_, our Δ_47_-based temperature record also enables us to assess the SST estimates from the other proxies and their underlying assumptions, both in terms of absolute temperatures and the total magnitude of warming (Fig. 4 and Table 2). In general, our biomarker-based reconstructions result in higher absolute temperatures for the studied interval than our foraminifera-based reconstructions. Although temperature proxies may disagree for a variety of reasons, it is likely that part of this difference is attributed to different proxies tracking different water depths and/or seasons. First, we note that saturated U^K′^_37_ values in modern sediments are observed at an SST range of 24° to 29°C and therefore do not always correspond to the 28°C limit of the U^K′^_37_-temperature calibration (*65*). Nevertheless, U^K′^_37_ may still be biased toward summer temperatures relative to the other proxies (*65*), particularly in the North Atlantic (*75*). GDGT fluxes governing TEX_86_ are expected to be greatest in the season of highest productivity (*76*), which, in the modern North Atlantic, is spring (*77*). However, no effect of seasonality is apparent in the TEX_86_-temperature calibration based on the modern core-top dataset (*64*) or sediment trap studies (*76*, *78*). Alternatively, GDGTs and alkenones accumulating at the Newfoundland Drifts could potentially be more strongly affected by lateral transport than planktonic foraminifera (*79*, *80*). However, because the source of North Atlantic drift sedimentation (*40*) is located toward the north of the study sites (i.e., in colder waters), lateral transport effects due to drift sedimentation would most likely result in a cold bias in biomarker-based temperatures rather than a warm bias (*66*), opposite to what we observe in our datasets. Nevertheless, differential lateral transport effects in the surface waters may be important (*81*) and could be exacerbated given the large (present-day) latitudinal temperature gradient offshore Newfoundland. Moreover, TEX_86_ and U^K′^_37_ are calibrated to reflect SST (i.e., 0-m water depth), while the planktonic foraminifera-based proxies reflect more integrated upper ocean mixed-layer conditions (*56*, *82*). We also explore subsurface TEX_86_ calibrations (*82*) for our dataset (fig. S8) and find that a subsurface calibration brings the TEX_86_-derived temperatures close to the Δ_47_-derived temperatures for *G. index*. However, it virtually eliminates all signs of warming for the MECO interval, which contrasts starkly with the *G. index* and bulk carbonate records. For comparison, structural offsets between absolute temperatures derived from TEX_86_, Mg/Ca, and δ^18^O_c_ have also been recognized in other Paleogene and older paleoclimate reconstructions (*4*) with similarly high uncertainties arising from non-analog and poorly constrained factors, including changes in seawater composition, species physiology, and taxonomy, among others.

**Table 2.** Comparison of multiproxy temperature reconstructions from Δ_47_, Mg/Ca, TEX_86_^H^, and U^K′^_37_ across the MECO warming, peak, and cooling phases. Temperatures are calculated as averages using the age definitions below. The Δ_47_-derived temperatures shown are the means listed in Table 1. Representative CIs for each record are discussed in the main text.

MECO phase	Age (Ma)	*A. (prae-)topilensis* temperatures		*A. bullbrooki* temperatures		*G. index* temperatures		Biomarker temperatures	
		Τ Δ_47_ (°C)	*T* Mg/Ca (°C)	Τ Δ_47_ (°C)	*T* Mg/Ca (°C)	Τ Δ_47_ (°C)	Τ Mg/Ca (°C)	Τ TEX_86_^H^ (°C)	Τ U^K′^_37_ (°C)
Cooling	<40.1	26.0	22.4	–	–	22.4	19.2	29.8	>28
Peak	40.1–40.3	26.4	24.5	–	–	25.3	19.7	31.2	>28
Warming	40.3–40.7	25.0	22.5	–	–	22.7	19.2	30.6	>28
Background	>40.7	23.0	22.4	21.6	–	–	–	29.7	>28

Furthermore, our Δ_47_-based estimates yield higher absolute temperatures and more pronounced temperature change across the MECO compared to Mg/Ca-based estimates derived from the same sets of foraminiferal specimens—in particular, for *G. index* (Fig. 3, B and C). Given the overall good-to-excellent quality of foraminiferal preservation in the studied interval (fig. S2), we consider it unlikely that our Δ_47_-derived temperatures are significantly biased toward lower values owing to some form of diagenetic alteration during burial (*47*, *83*). Solid-state clumped isotope reordering could theoretically change Δ_47_ without affecting δ^18^O_c_, but this is highly unlikely given the shallow burial depth of the study sites (*84*) and would result in warmer rather than cooler reconstructed temperatures. Moreover, a recent study testing the effects of diagenetic alteration on middle Eocene planktonic foraminiferal Δ_47_ from the Newfoundland Drifts (Sites U1408, U1409, and U1410) found no detectable impact of diagenetic alteration (*47*). Selective dissolution of Mg from foraminiferal tests could result in Mg/Ca-based temperatures that are biased toward cooler temperatures (*85*), particularly in the interval of very low carbonate content toward the peak of the MECO between 40.1 and 40.3 Ma. Evaluation of coupled Δ_47_ and Mg/Ca measurements relative to the Δ_47_-Mg/Ca relationships expected from their respective temperature calibrations (*86*) could be a way to resolve this, but we cannot make definitive inferences owing to the limited material available for paired measurements in high resolution and the uncertainties involved in Mg/Ca-temperature reconstructions (Mg/Ca-temperature relationships, salinity, pH, and Mg/Ca_sw_; see Materials and Methods). Moreover, any transient seawater salinity increase or pH decrease during the MECO would reduce the total amount of warming inferred from Mg/Ca. Although the relative warming inferred from Δ_47_ and Mg/Ca for *A. (prae-)topilensis* are in fairly good agreement with each other in those parts of the record where this species is present (Fig. 3B), the absence of any increase in Mg/Ca for *G. index* during the MECO lies in stark contrast with the warming inferred from Δ_47_ for *G. index* (Fig. 3C), which remains regardless of the calibration chosen. Collectively, our records show that the set of confounding uncertainties discussed above may result in rather low agreement between the different paleotemperature proxies, which highlights the critical importance of integrated multiproxy reconstructions and balanced data evaluation. Until a fully integrated inversion workflow for temperature proxies is established, we recommend that future paleoclimate studies incorporate Δ_47_ in addition to all other data types available within the constraints of the local oceanographic and sedimentary environments.

## MATERIALS AND METHODS

### Site descriptions

International Ocean Discovery Program (IODP) Sites U1408 and U1410 are both located on the southeast Newfoundland Ridge in the northwest Atlantic Ocean (*39*). Site U1408 is situated at 41°26′N, 49°14′W (~3022 m in water depth), and Site U1410 is situated at 41°20′N, 49°10′W (~3400 m in water depth) (*39*, *48*, *49*). The paleodepth for Sites U1408 and U1410 at 50 Ma is estimated at ~2575 and ~2950 m below sea level, respectively (*48*, *49*). Both sites contain sequences of calcareous middle Eocene sediments deposited above the carbonate compensation depth (CCD), but the shallower Site U1408 sediments are especially enriched in carbonate and are more expansive than the sediments of Site U1410 (*48*, *49*). The MECO interval at Site U1408 consists of alternations between greenish gray nannofossil clays and whitish to light gray nannofossil oozes, which feature a well-developed cyclicity on a decimeter scale and a higher carbonate content than underlying and overlying sediments (*48*). Because a hiatus was recognized in the top part of the MECO interval at Site U1408, we incorporate an additional interval from Site U1410 to construct a composite record that captures the full extent of the MECO.

### Stratigraphy and age models

An astronomically tuned age model is available for Site U1408 and U1410 sediments (*54*), but reevaluation of the available X-Ray Fluorescene (XRF) data paired with recently generated bulk carbonate and benthic foraminiferal stable isotope records shows that the peak warming interval at Site U1408 actually contains a hiatus (see fig. S3) (*41*). Hence, we use a revised composite depth scale (CCSF-X) based on a tentative reinterpretation of the available data and tie that to the established astrochronology, as presented in (*41*). See table S1 for an overview of the tie points used. We note that these stratigraphic correlations could be further improved at a later stage if even more complete datasets were to become available.

### Sampling and foraminiferal species selection

The MECO interval was sampled at a continuous, high resolution (~3 cm) at Site U1408. In addition, a smaller sample set of similar resolution was taken from the inferred MECO peak warming interval at Site U1410. Samples were washed and sieved, and subsequently, specimens of mixed-layer dwelling planktonic foraminifera were picked from the 250- to 355-μm size fraction for three species (groups): *A. (prae-)topilensis*, *A. bullbrooki*, and *G. index*.

### Foraminiferal preservation

Foraminiferal preservation is generally good to excellent at Sites U1408 and U1410, owing to the high clay content of the sediments deposited in the Newfoundland Drifts (*48*, *49*). Many foraminiferal shells at Site U1408 have glassy textures (fig. S2), which is regarded as an exceptional preservation state for foraminifera and allows for geochemical reconstructions of original seawater conditions with little to no diagenetic alteration (*51*, *87*). A similar high quality of foraminiferal preservation has also been reported for Late Eocene sediments at Site U1411 (*88*), which is located in close proximity to Sites U1408 and U1410.

### Cleaning

Shells of the aforementioned planktonic foraminiferal species were cracked and cleaned following the protocol of (*89*). For clumped and stable isotope analyses, this involved the cracking of at least 20 foraminiferal shells per sample between two glass plates and the removal of clays and coccoliths through ultrasonication in 50 μl of deionized water, followed by treatment with 200 μl of methanol (CH_3_OH) and three additional rinsing steps with 200 μl of deionized water. For trace element analyses, splits of methanol-cleaned and homogenized material were subjected to further cleaning involving the removal of organic matter through oxidative treatment in hot alkali-buffered hydrogen peroxide (H_2_O_2_) and the removal of adsorbed contaminants through weak acid leaching with nitric acid (HNO_3_). To minimize sample loss, a reductive treatment step was omitted.

### Clumped and stable isotope analyses

The clumped isotope paleothermometer (Δ_47_) is defined as the excess of the mass-47 CO_2_ isotopologues relative to their expected abundance in a stochastic distribution of the ^13^C and ^18^O isotopes among all isotopologues (*44*). Cleaned and homogenized shells of *A. (prae-)topilenis*, *A. bullbrooki*, and *G. index* were analyzed for clumped (Δ_47_) and stable (δ^18^O_c_ and δ^13^C_c_) isotopic compositions at Utrecht University (UU) and the University of Bergen (UiB). We analyzed one to three aliquots per sample to generate high-resolution Δ_47_ and δ^18^O_c_/δ^13^C_c_ isotope records simultaneously, with a total of 365 samples and 514 analyses. Samples analyzed at UU consist of *A. (prae-)topilenis*, *A. bullbrooki*, and *G. index* from the entire studied interval of Sites U1408 and U1410, while samples analyzed at UiB consist of *G. index* from the peak-MECO interval of Site U1408 and *A. (prae-)topilenis* from the onset of MECO warming at Site U1408 (fig. S5). Excellent agreement was observed in a subset of samples that were analyzed at both labs.

At UU, measurements were performed on two instruments, using either a Thermo Fisher Scientific MAT 253 mass spectrometer or a Thermo Fisher Scientific 253 Plus mass spectrometer, respectively, coupled to a Thermo Fisher Scientific Kiel III or Kiel IV carbonate device. Consequently, the analytical methods differed between the two instruments. Approximately 120 to 180 μg of material was used per measurement on the MAT 253 and approximately 80 to 100 μg per measurement on the 253 Plus, due to differences in the sensitivities of both mass spectrometers. Samples were reacted with hypersaturated phosphoric acid (H_3_PO_4_) at 70°C; in the Kiel IV setup, the CO_2_ from these samples was passed through a PoraPak Q trap kept at −40°C to filter out any organic contaminants (*90*), whereas in the Kiel III setup, a Peltier cooling system kept at −20°C was used instead. During each instrument sequence, 16 to 20 aliquots of randomly selected samples were analyzed alongside multiple replicates of the ETH-1, ETH-2, ETH-3, ETH-4, and IAEA-C2 carbonate standards (respectively named after the Eidgenössische Technische Hochschule Zürich and International Atomic Energy Agency institutes). The analyses on the Thermo Fisher Scientific MAT 253 mass spectrometer consisted of eight measurement cycles, switching between sample and reference, while the analyses on the Thermo Fisher Scientific 253 Plus mass spectrometer used the long-integration dual-inlet (LIDI) method (*91*). During LIDI analyses, sample gas was measured continuously for 400 s, followed by the reference gas for 410 s. At UiB, measurements were performed on two different Thermo Fisher Scientific 253 Plus mass spectrometers coupled to Thermo Fisher Scientific Kiel IV carbonate devices using similar methods as at UU (LIDI technique with 400 s of integration time; ETH-1, ETH-2, and ETH-3 carbonate standards for data correction; acid digestion at 70°C; PoraPak Q temperature at −20°C).

### Clumped isotope data processing

For the 253 Plus instruments at UU and UiB, a pressure-baseline (PBL) correction (*92*, *93*) was performed using daily background scans (high voltage versus beam intensity on different masses). Briefly, a linear regression between the minima in the signal plateau left of the peak of masses 45 to 48 and the maxima of mass 44 was performed, resulting in a mass-44 intensity–dependent correction for secondary electron backscatter. At UU, a factor of 0.9 was applied to the PBL correction based on improved final values of standards ETH-1, ETH-2, and Merck (Δ_47_ values, long-term SDs, and offsets). Background scans were also performed on the MAT 253 mass spectrometer, but a factor of 0.76 was used for the Kiel III setup compared to a factor of 0.9 for the Kiel IV setup. Raw Δ and δ values were calculated using the International Union of Pure Applied Chemistry (IUPAC) parameters. All Δ_47_ measurements were transferred to the Absolute Reference Frame or Intercarb Carbon Dioxide Equilibrated Scale (I-CDES) by calculating the empirical transfer function (ETF) based on the carbonate standards ETH-1, ETH-2, and ETH-3 using their values reported in (*46*).

For samples measured at UU, data analysis was performed in R (*94*) using the packages *isoreader* and *clumpedr*. Automatic detection of failed measurements was performed on the basis of sudden pressure drops during acquisition, contamination estimates from mass 49, initial sample, and reference gas intensities (intensities outside of 8 to 40 V range or with >3-V difference between sample and reference gas were excluded) and final Δ_47_ values of analyzed standards (>4SD off deemed outlier). Standards and samples were corrected for intrarun drift by subtracting a rolling average of ETH-3 with a width of 7 aliquots for Δ_47_ and of ETH-1, ETH-2, and ETH-3 with a width of 15 aliquots for δ^18^O_c_ and δ^13^C_c_. Conversion to the absolute reference frame was performed with a 201-aliquot-rolling ETF that spanned multiple preparations.

For samples measured at UiB, clumped isotope data were processed using Easotope (*95*), including a PBL correction based on daily background scans (without scaling factor), and a rolling ETF interval of in total 20 to 60 separate standard measurements (ETH-1, ETH-2, and ETH-3) before and after each sample measurement. The number of standards measurements used for correction was determined on the basis of instrument stability. δ^18^O_c_ and δ^13^C_c_ values were corrected for drift using a total of 60 standard measurements of ETH-1, ETH-3, and either ETH-2 or ETH-4.

Replicate measurements of the IAEA-C2 (UU) and ETH-4 (UiB) standards were used to estimate the external reproducibility of Δ_47_, δ^18^O_c_, and δ^13^C_c_. On the basis of 90-replicate IAEA-C2 measurements at UU over the full length of the study period, external reproducibility (1σ) is estimated at 0.0365‰ for Δ_47_, 0.07‰ for δ^18^O_c_, and 0.03‰ for δ^13^C_c_. At UiB, external reproducibility was 0.0354‰ for Δ_47_, 0.06‰ for δ^18^O_c_, and 0.03‰ for δ^13^C_c_ based on 606 measurements of ETH-4 during the study period. δ^18^O_c_ and δ^13^C_c_ values are reported relative to the Vienna Pee Dee belemnite (VPDB) standard. See fig. S5 for an overview of all raw Δ_47_ data for Sites U1408 and U1410 and the laboratories where each replicate was measured.

### Temperature reconstructions using Δ_47_

We calculate Δ_47_-based temperatures using the updated foraminifera-based Δ_47_-based temperature calibration of (*96*), based on the I-CDES scale as proposed by (*46*). This is expressed in the following two equations

$$\Delta_{47}=0.0397(\pm 0.0011)∙\frac{10^{6}}{T^{2}}+0.1518(\pm 0.0128)$$ (1)

$$T=\sqrt{\frac{0.0397(\pm 0.0011)∙10^{6}}{\Delta_{47}-0.1518(\pm 0.0128)}}$$ (2)

where Δ_47_ is given in ‰ (I-CDES) and *T* represents the temperature in kelvin.

We use a combination of two data analysis approaches to converting our Δ_47_ measurements to temperature estimates (*97*). The first approach is based on averaging sets of Δ_47_ replicates from neighboring samples to obtain precise temperature estimates. For this purpose, we select clusters of at least 20 but ideally >40 neighboring measurements per foraminiferal species group in prescribed time intervals of 0.1 to 0.2 Ma. We calculate mean Δ_47_ values for each cluster and report them with their respective 95% CIs, based on the SD of the included measurements and the number of included measurements. This approach generally results in CIs that are larger than the external reproducibility inferred from the IAEA-C2 standard. The mean Δ_47_ values and confidence margins of these clusters are subsequently converted to temperature using Eq. 2. The age range of the clusters incorporates the full age range of the included measurements.

The second approach that we use to obtain temperature estimates is based on LOESS, which we apply to all Δ_47_ values per foraminiferal species group. We calculate the 2.5th, 16th, 50th, 84th, and 97.5th percentiles of these LOESS fits to obtain the median Δ_47_ estimates and their associated 68 and 95% CIs, which are subsequently converted to temperature using Eq. 2. For simplicity, we ignore the uncertainties associated with the Δ_47_-based temperature calibration, because these calibration uncertainties (± 1°C at 95% CI) are much smaller than the typical analytical uncertainties for foraminifer-based Δ_47_ estimates (*93*, *98*).

### Seawater δ^18^O and salinity reconstructions

Our foraminiferal δ^18^O records (δ^18^O_c_) and Δ_47_-based temperature records enable us to estimate changes in the δ^18^O composition of seawater (δ^18^O_sw_) across the MECO. To this end, we use the δ^18^O_c_-temperature calibration of (*99*) as modified by (*100*), which is based on precipitation of synthetic calcite at a temperature range of 10° to 40°C and is recommended by DeepMIP for Paleogene reconstructions (*4*). It is expressed as follows

$$T=16.1-4.64∙(\delta^{18}O_{c}-\delta^{18}O_{\mathrm{sw}})+0.09∙{\left(\delta^{18}O_{c}-\delta^{18}O_{\mathrm{sw}}\right)}^{2}$$ (3)

where *T* is temperature expressed in degrees Celsius, δ^18^O_c_ in ‰ (VPDB), and δ^18^O_sw_ in ‰ [Vienna standard mean ocean water (VSMOW)].

First, we calculate mean δ^18^O_c_ values for each of the aforementioned Δ_47_ clusters per foraminiferal species group, using the same prescribed time intervals. We then use the mean δ^18^O_c_ values and mean Δ_47_-derived temperatures for each cluster to solve Eq. 3 and obtain mean δ^18^O_sw_ estimates. Because the CIs for δ^18^O_c_ are much smaller than the CIs in Δ_47_-derived temperatures for each cluster, we only use the CIs of the latter and propagate them to provide CIs for δ^18^O_sw_.

In addition, we calculate individual δ^18^O_sw_ estimates for all paired Δ_47_ and δ^18^O_c_ measurements and subsequently fit an LOESS to those δ^18^O_sw_ estimates from all foraminiferal species groups combined. We then calculate the 2.5th, 16th, 50th, 84th, and 97.5th percentiles of these LOESS fits to obtain the median δ^18^O_sw_ estimates and their associated 68 and 95% CIs.

Last, we use our δ^18^O_sw_ records to reconstruct changes in sea surface salinity across the MECO. For this purpose, we use the modern δ^18^O_sw_-salinity relationship for the Atlantic Ocean (*101*, *102*) to obtain a first-order approximation of absolute salinity. Salinity (*S*) is calculated as follows

$$\delta^{18}O_{\mathrm{sw}}=-19.264+0.558∙S$$ (4)

$$S=\frac{\delta^{18}O_{\mathrm{sw}}+19.264}{0.558}$$ (5)

The slope of this modern δ^18^O_sw_-salinity relationship (0.558‰/psu) for the Atlantic Ocean is similar to the conservative estimate of 0.50‰/psu used in salinity reconstructions for the PETM (*6*) and ETM-2 (*18*). However, because δ^18^O_sw_-salinity relationships are also affected by temperature and the δ^18^O value of precipitation (*67*), the exact expression of this relationship for the middle Eocene remains uncertain. Therefore, we prefer to present our estimates as salinity anomalies relative to δ^18^O_sw_ = 0‰.

### Trace element analyses

Splits of fully cleaned and homogenized shells of *A. (prae-)topilenis* and *G. index* were analyzed for trace elemental compositions at the Royal Netherlands Institute for Sea Research (NIOZ) using a Thermo Fisher Scientific Element 2 inductively coupled plasma mass spectrometer (ICP-MS). Samples were dissolved in 0.1 M HNO_3_ and were diluted to a Ca^2+^ concentration of 40 parts per million following an initial run to determine optimal Ca concentrations. Quantitative Mg/Ca ratios were calculated against five ratio calibration standards with similar matrix using a ratio calibration method (*103*). Monitor standards JCp-1 *Porites* spp. and JCt-1 *Tridacna gigas* were measured to monitor the accuracies. Drift corrections were applied to improve the accuracy and were based on replicate analyses of an in-house coral monitor standard. Standard analytical precision at NIOZ is estimated to be better than ±0.4%.

The efficiency of our cleaning protocol and potential diagenetic effects on the resulting Mg/Ca ratios were assessed by evaluation of the corresponding Fe/Ca, Mn/Ca, Al/Ca, and Sr/Ca ratios (fig. S6). We have excluded measurements that displayed high Fe/Ca ratios (>0.8 mmol/mol for *A. (prae-)topilensis* and >0.5 mmol/mol for *G. index*; cutoffs were chosen relative to the values recorded by each species group) or Al/Ca ratios (>0.25 mmol/mol for both species groups) compared to the rest of the dataset but find no substantial evidence for contamination from detrital phases. We observe several *A. (prae-)topilensis* measurements with low Sr/Ca ratios relative to the rest of the dataset, which could hint at signs of diagenetic Sr and Mg loss, but these outliers are automatically excluded on the basis of the aforementioned criteria for Fe/Ca.

### Temperature reconstructions using Mg/Ca

Here, we primarily adopt the calibration of (*60*) to convert our foraminiferal Mg/Ca values (Mg/Ca_foram_) to absolute temperature estimates but we also explore alternatives below. This calibration for *Globigerinoides ruber* takes changes in both salinity (*S*) and the carbonate system (either through pH or [CO_3_^2−^]) into account and arrives at a different, more robust Mg/Ca-temperature relationship than the canonical calibration of (*104*), due to the use of climatological temperature estimates rather than δ^18^O_c_-based temperature estimates. Consequently, the calibration of (*60*) yields significantly lower temperatures for a given Mg/Ca_foram_ value. Here, we use their calibration for pH but with modifications to account for differences in the Mg/Ca composition of seawater (Mg/Ca_sw_) between past and present-day conditions based on (*61*). The resulting expression is

$$T=\frac{1}{0.060}[\ln\left(\mathrm{Mg}/{\mathrm{Ca}}_{\mathrm{foram}}\frac{{\left[\mathrm{Mg}/{\mathrm{Ca}}_{\mathrm{sw},t_{0}}\right]}^{H}}{{\left[\mathrm{Mg}/{\mathrm{Ca}}_{\mathrm{sw},t}\right]}^{H}}\right)-0.033∙S+0.83∙(\mathrm{pH}-8.0)+1.07]$$ (6)

where *T* represents temperature in degrees Celsisus, Mg/Ca_sw,*t*_0__ and Mg/Ca_sw,*t*_ respectively represent the Mg/Ca compositions of present-day and past seawater, and *H* represents the power component of the relationship between Mg/Ca_foram_ and Mg/Ca_sw_ following (*61*).

We calculate absolute temperatures with full propagation of the associated uncertainties using Monte Carlo simulations (*n* = 1000). These simulations incorporate analytical uncertainty and uncertainty in salinity, pH, and Mg/Ca_sw_. Considering the lack of independent salinity reconstructions for the middle Eocene and the strong present-day salinity gradient at the study sites (fig. S1), we assume a relatively large range of possible salinity values between 32 and 36 psu (uniform distribution). For pH, we assume values between 7.6 and 8.0 (uniform distribution) to reflect a range of probable middle Eocene estimates from both modeling studies (*105*) and reconstructions based on boron isotopic compositions (δ^11^B) of planktonic foraminifera (*12*, *106*). For Mg/Ca_sw,*t*_, we assume values between 1.5 and 3.0 mmol/mol for the middle Eocene, which is relatively well constrained on the basis of a range of reconstructions from fluid inclusions (*107*), calcium carbonate veins (*108*), and large benthic foraminifera (*109*); for Mg/Ca_sw,*t*_0__, we use the present-day value of 5.2 mmol/mol. Last, for *H*, we use a value of 0.42 based on best estimates for planktonic foraminifera based on *Globigerinoides sacculifer* (*110*, *111*). Following the Monte Carlo simulations based on these parameters, we applied LOESS fitting to every iteration of our dataset and calculated the 2.5th, 16th, 50th, 84th, and 97.5th percentiles of these LOESS fits to obtain the median temperature estimates and their associated 68 and 95% CIs.

In addition to our Monte Carlo simulations of Mg/Ca-based temperatures, we also present an optimal scenario based on *S* = 34 psu, pH = 7.8, and Mg/Ca_sw_ = 2.25 mmol/mol (Fig. 4C). We do not explicitly account for an increase in salinity as obtained from our Δ_47_-based reconstructions (Fig. 3B) or a potential decrease in pH during the MECO resulting from CO_2_ rise and ocean acidification (*11*, *12*, *22*), but note that both of these factors would result in a reduction in the total amount of inferred warming. For our observed range of Mg/Ca_foram_ values, a progressive salinity increase from 34 to 36 psu would reduce total warming by ~1°C, while a progressive pH decrease from 7.8 to 7.6—as reported for the peak MECO interval by (*12*)—would reduce total warming by ~3°C. Given the long oceanic residence times of Mg and Ca, we assume no transient change in Mg/Ca_sw_ across the MECO.

Although we are able to account reasonably well for the uncertainties regarding both pH and Mg/Ca_sw_, the exact expression of the Mg/Ca-temperature calibration remains the largest source of uncertainty for deep-time Mg/Ca-based temperature reconstructions. Therefore, we also perform our calculations with two different Mg/Ca-temperature relationships (fig. S7). First, we followed the Mg/Ca-temperature relationships outlined in (*109*), which are based on the (*104*) calibration and account for changes in both pH (*112*) and Mg/Ca_sw_ (*113*). These Mg/Ca-temperature relationships have been used by (*109*) to recalculate existing Mg/Ca-derived temperatures for Eocene planktonic foraminifera and are expressed as

$$T=\frac{1}{A}\ln\left(\frac{\mathrm{Mg}/{\mathrm{Ca}}_{\mathrm{norm}}}{B}\right)$$ (7)

$$\mathrm{Mg}/{\mathrm{Ca}}_{\mathrm{norm}}=\frac{\mathrm{Mg}/{\mathrm{Ca}}_{\mathrm{foram}}}{\frac{0.66}{1+\exp(6.9∙(\mathrm{pH}-8.0))}+0.76}$$ (8)

$$B=0.019∙\mathrm{Mg}/{{\mathrm{Ca}}_{\mathrm{sw}}}^{2}-0.16∙\mathrm{Mg}/{\mathrm{Ca}}_{\mathrm{sw}}+0.804$$ (9)

$$A=-0.0029∙\mathrm{Mg}/{{\mathrm{Ca}}_{\mathrm{sw}}}^{2}+0.032∙\mathrm{Mg}/{\mathrm{Ca}}_{\mathrm{sw}}$$ (10)

For a direct comparison of temperature estimates we again use pH = 7.8 and Mg/Ca_sw_ = 2.25 mmol/mol, similar to the scenario outlined above for the calibration of (*60*).

Second, we test the widely applied calibration of (*104*), with modifications for changing Mg/Ca_sw_ following (*61*). This Mg/Ca-temperature relationship has, for instance, been used for foraminiferal Mg/Ca records of the PETM (*8*, *111*) and is expressed as

$$T=\frac{1}{A}\ln\left(\frac{\mathrm{Mg}/{\mathrm{Ca}}_{\mathrm{foram}}}{B}\frac{{\left[\mathrm{Mg}/{\mathrm{Ca}}_{\mathrm{sw},t_{0}}\right]}^{H}}{{\left[\mathrm{Mg}/{\mathrm{Ca}}_{\mathrm{sw},t}\right]}^{H}}\right)$$ (11)

We use the default values of *A* = 0.09 and *B* = 0.38 following (*104*), as well as Mg/Ca_sw,*t*_ = 2.25 mmol/mol, Mg/Ca_sw,*t*_0__ = 5.2 mmol/mol, and *H* = 0.42 for consistency.

These alternative Mg/Ca-temperature relationships both yield remarkably similar temperature estimates for our observed range of Mg/Ca_foram_ values (fig. S7), but the resulting temperatures are ~5°C higher than those obtained from our preferred Mg/Ca-temperature calibration of (*60*). This major difference has implications for previous Mg/Ca-based temperature records that are calculated with the calibration of (*104*).

### Biomarker paleothermometry

For a total of 53 samples from Sites U1408 and U1410, lipids were extracted from 4 to 6 g of freeze-dried and powdered sediment with dichloromethane (DCM):methanol (MeOH; 9:1, v/v) using a Dionex accelerated solvent extractor (ASE 350) at a temperature of 100°C and a pressure of 7.6 × 10^6^ Pa. Lipid extracts were separated into an apolar, ketone, and polar fraction by Al_2_O_3_ column chromatography using hexane:DCM (9:1), hexane:DCM (1:1), and DCM:MeOH (1:1) as respective eluents. Ninety-nine grams of a synthetic C_46_ [mass/charge ratio (*m*/*z*) = 744] glycerol trialkyl glycerol tetraether standard was added to the polar fraction, which subsequently was dissolved in hexane:isopropanol (99:1, v/v) to a concentration of ~3 mg/ml and passed through a 0.45-μm polytetrafluoroethylene filter. This fraction was then analyzed by high-performance liquid chromatography (HPLC) and atmospheric pressure chemical ionization mass spectrometry using an Agilent 1260 Infinity series HPLC system coupled to an Agilent 6130 single-quadrupole mass spectrometer at UU following (*114*) to measure the abundance of GDGTs. The BIT index and TEX_86_ values were calculated according to (*115*, *116*), respectively, although the BIT index was adjusted to include both 5- and 6-methyl brGDGTs. Temperatures were calculated with the logarithmic TEX_86_^H^ calibration, which has an associated calibration uncertainty of ~2.5°C (*63*). The long-term analytical precision for TEX_86_ at UU based on replicate analyses of an in-house standard is ±0.3°C.

We use established indicator ratios between different isoGDGTs to assess GDGT sourcing by microbes other than surface ocean-dwelling marine Thaumarchaeota, which might overprint the temperature signal recorded in TEX_86_. We calculate the Methane Index (*117*) (cutoff value, 0.3) and assess the GDGT-0/crenarchaeol (*118*) (cutoff value, 2.0) and GDGT-2/crenarchaeol (*119*) (cutoff value, 0.2) ratios to respectively evaluate contributions by methanotrophic-, methanogenic-, and AOM (anaerobic oxidation of methane)–associated archaea. The GDGT-2/GDGT-3 ratio (cutoff value, 5.0) is used to assess GDGT production by deeper-dwelling archaea (*120*). Aberrant proportions of the crenarchaeol isomer are quantified as Cren′ relative to (Cren′ + Cren) with a cutoff value of 0.25 (*121*). The isoGDGT distribution as a whole is evaluated by its deviation from the modern pelagic TEX_86_-to-Ring Index relationship in the ΔRI (cutoff value, |0.3|) (*122*). Last, the BIT index is used to indicate the relative dominance of branched GDGTs over isoGDGTs.

All analyzed samples yielded sufficient GDGTs for integration. While BIT values are very high throughout the studied interval (0.69 ± 0.11, 1σ), we find no distinct relationship between BIT and TEX_86_ (fig. S8), suggesting that the relatively high supply of branched GDGTs was not accompanied by the influx of anomalous isoGDGTs affecting TEX_86_. Half of the studied samples (10 of 28 for Site U1408; 16 of 24 for Site U1410) suffer from ΔRI values of >0.3 (figs. S9 and S10), indicating that these middle Eocene isoGDGT distributions have a large offset from the modern global TEX_86_-RI relationship. The TEX_86_ values of these samples are thus likely to have been affected by other factors than SST. Many of these samples also have elevated GDGT-2/Cren ratios of >0.2, suggesting a possible contribution by AOM-associated archaea (fig. S9). On the basis of these observations, we exclude samples with aberrant values based on the RI and other isoGDGT ratios but not based on the BIT index. Last, the composition of the brGDGTs and, in particular, the low #rings_tetra_ index values (0.08 ± 0.01, 1σ), suggests that they are soil-derived (*123*).

Furthermore, 15 ketone fractions were analyzed for alkenones at NIOZ using an Agilent 6890 N gas chromatograph (GC) with flame ionization detection after dissolving in ethyl acetate. The GC was equipped with a fused silica column with a length of 50 m, a diameter of 0.32 mm, and a coating of CP Sil-5 (film thickness, 0.12 μm). Helium was used as carrier gas, and the flow mode was a constant pressure of 100 kPa. The ketone fractions were injected on-column at a starting temperature of 70°C, which increased by 20°C/min to 200°C, followed by 3°C/min until the final temperature of 320°C was reached. This end temperature was held for 25 min. Selected fractions were analyzed by GC–mass spectrometry using an Agilent 7890B GC coupled to an Agilent 5977A mass spectrometer. The starting temperature was 70°C and increased to 130°C by 20°C/min, followed by a linear gradient of 4°C/min to an end temperature of 320°C, which was held for 25 min. One microliter was injected, and separation was achieved on a fused silica column (25 m by 0.32 mm) coated with CP Sil-5 (film thickness, 0.12 μm). Helium was used as carrier gas with a constant flow of 2 ml/min. The mass spectrometry operated with an ionization energy of 70 eV. Identification of alkenones was done in full scan mode, scanning between 50 and 850 *m*/*z*.

Alkenones could be detected in most analyzed ketone fractions. However, these alkenones consisted only of the C_37:2_ and C_38:2_ alkenones, yielding U^K′^_37_ = 1 (proxy saturation) and hence SSTs of at least 28°C (*65*).

## Supplementary Materials

### This PDF file includes:

Figs. S1 to S10 Table S1 References
