# Supplementary material for: North Atlantic surface ocean warming and salinization in response to middle Eocene greenhouse warming
Source: Sci Adv. 2023 Jan 25;9(4):eabq0110. doi: 10.1126/sciadv.abq0110 (PMC9876553; doi:10.1126/sciadv.abq0110)
Supplement: Supplementary file 1 — Figs. S1 to S10 Table S1 References [file sciadv.abq0110_sm.pdf]

Science Advances
AAAS

Supplementary Materials for

North Atlantic surface ocean warming and salinization in response to middle Eocene greenhouse warming

Robin van der Ploeg *et al.*

Corresponding author: Robin van der Ploeg, r.vanderploeg@shell.com

Sci. Adv. **9**, eabq0110 (2023)
DOI: 10.1126/sciadv.abq0110

The PDF file includes:

Figs. S1 to S10
Table S1
Legends for data S1 to S4
References

Other Supplementary Material for this manuscript includes the following:

Data S1 to S4

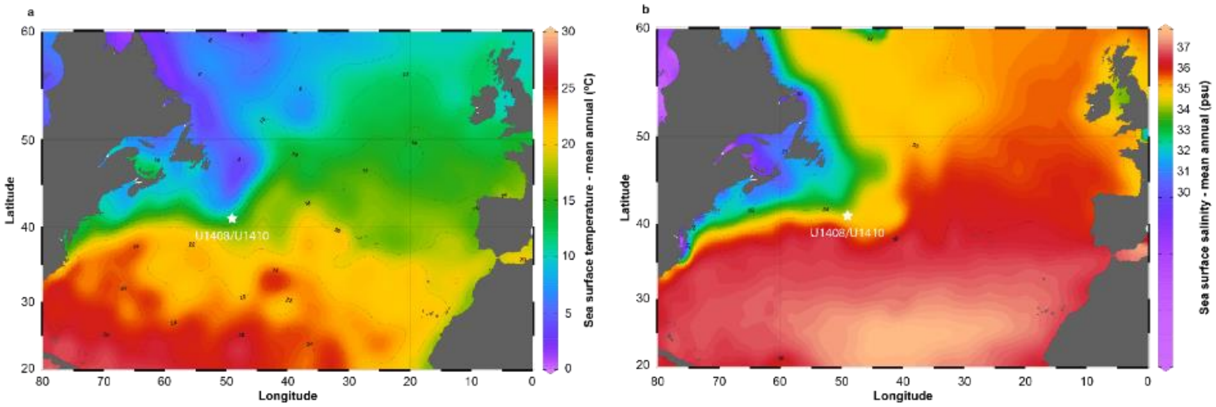

Fig. S1: Present-day North Atlantic sea surface conditions.

a, Mean annual sea surface temperature. **b,** Mean annual sea surface salinity. Map made with Ocean Data View (*126*). The locations of Sites U1408 and U1410 are indicated by the white star.

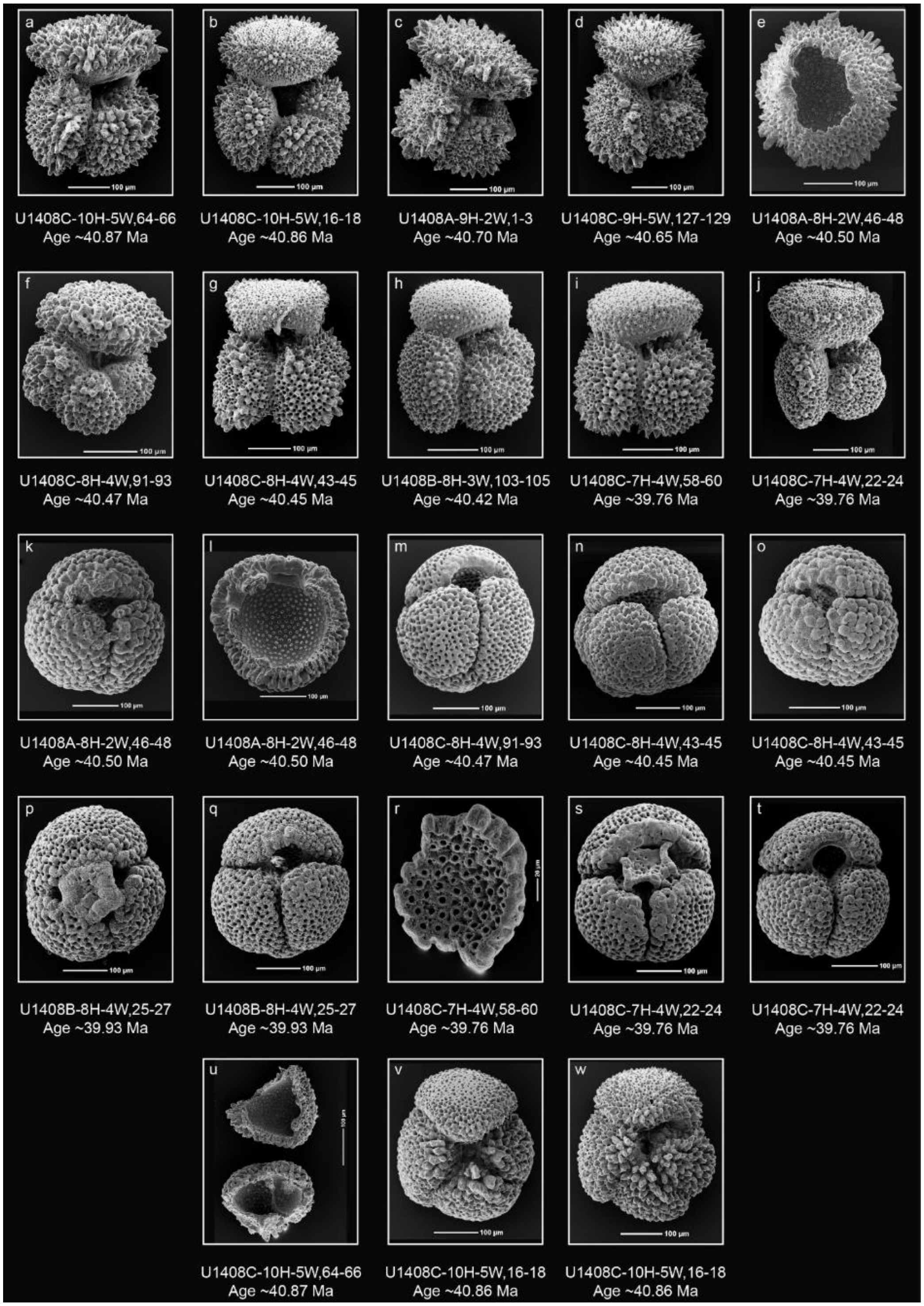

Fig. S2: Scanning electron microscope (SEM) images of representative specimens of planktonic foraminifera from the studied interval at Site U1408.

a-j, *A. (prae-)topilensis*, **k-t,** *Gk. index*. **u-w**, *A. bullbrooki*. Sample information and the corresponding ages following our revised composite age model for U1408 and U1410 are included.

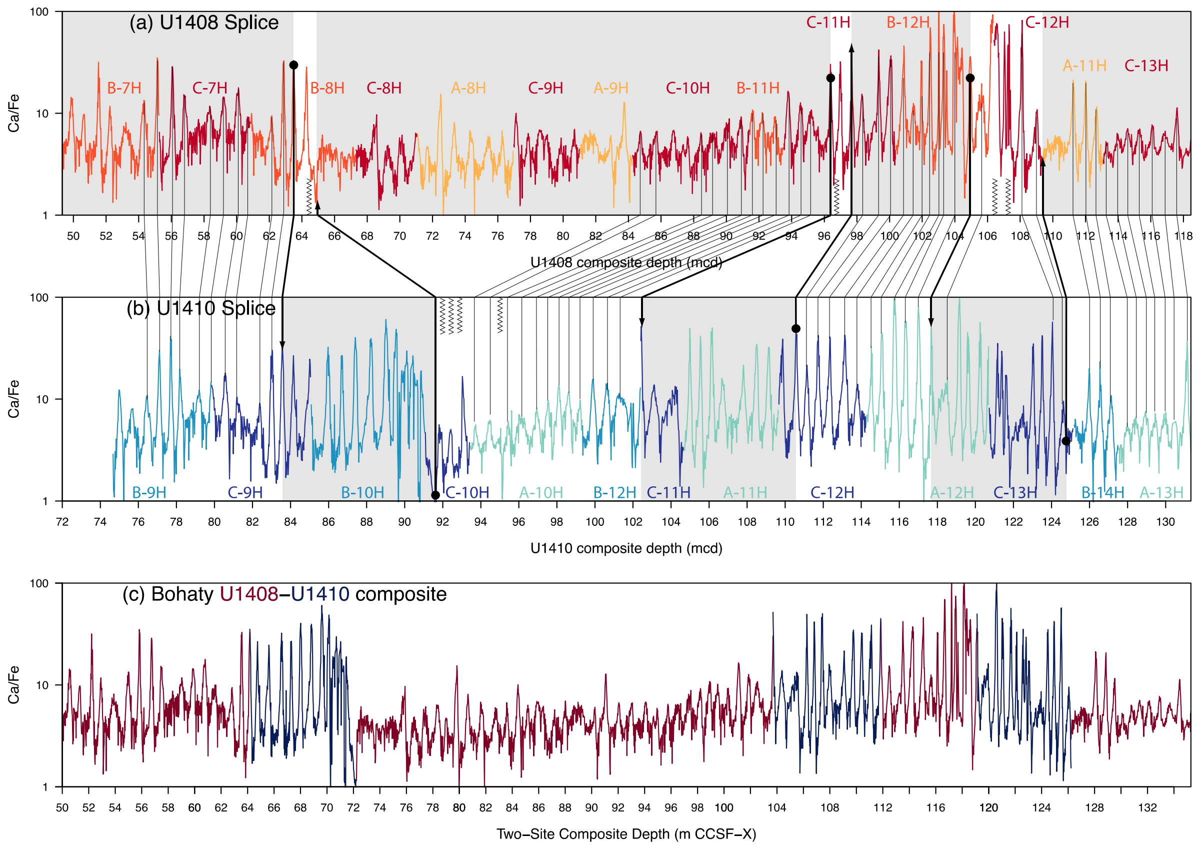

Fig. S3: Revised composite stratigraphy for Sites U1408 and U1410.
Ca/Fe data and site correlations as presented in Figure 3 of (*41*).

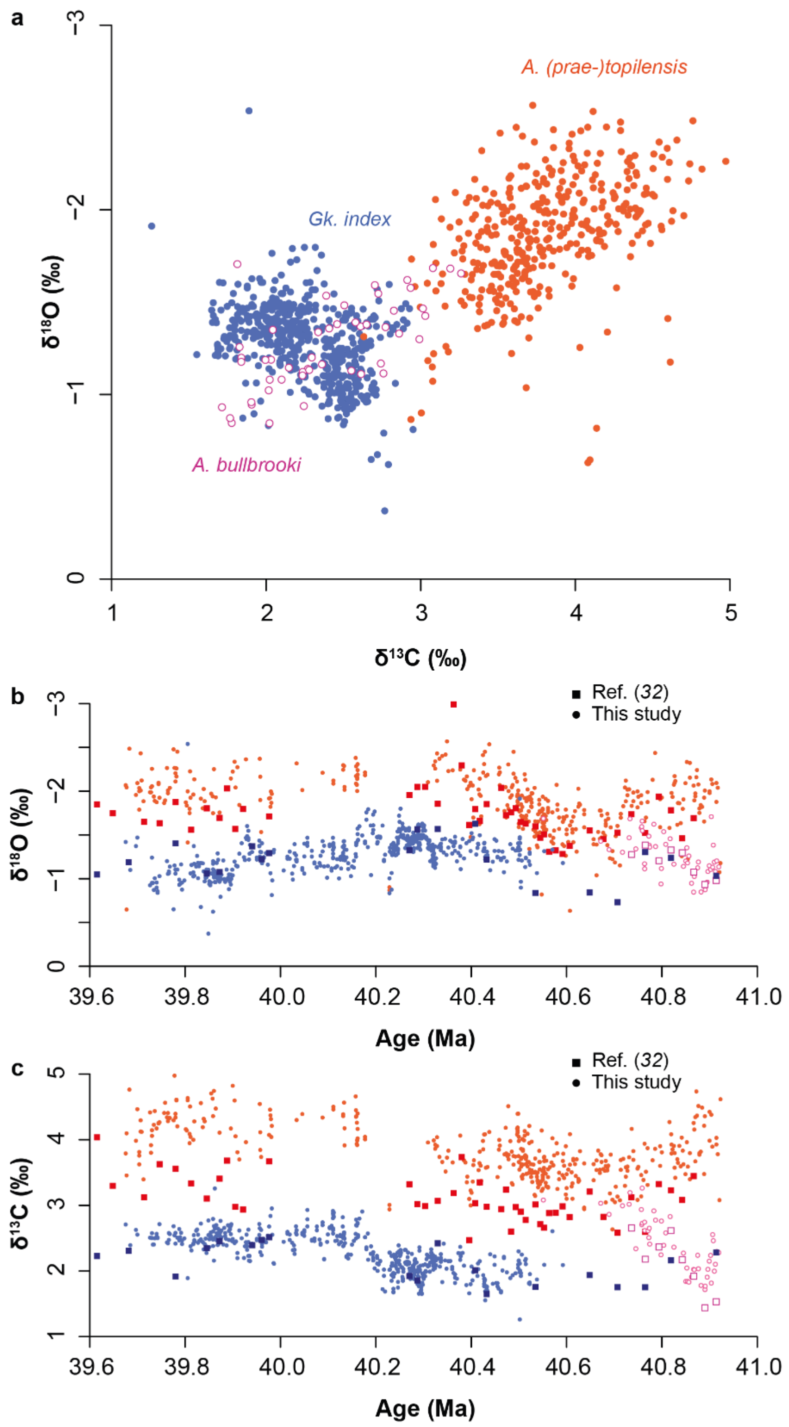

Fig. S4: $\delta^{18}O_c$ and $\delta^{13}C_c$ relationships for the foraminiferal species groups included in this study and comparison to previously published Site U1408 records.

a, $\delta^{18}O_c$ and $\delta^{13}C_c$ data for *A. (prae-)topilensis* (in red), *A. bullbrooki* (in purple) and *Gk. index* (in blue) from Sites U1408 and U1410 (this study). **b-c,** Comparison between the $\delta^{18}O_c$ and $\delta^{13}C_c$ data presented in this study (circles; colors as in (**a**)) and previously published data for the same foraminiferal species groups from the MECO at Site U1408 by (*32*) (squares; in darker shades).

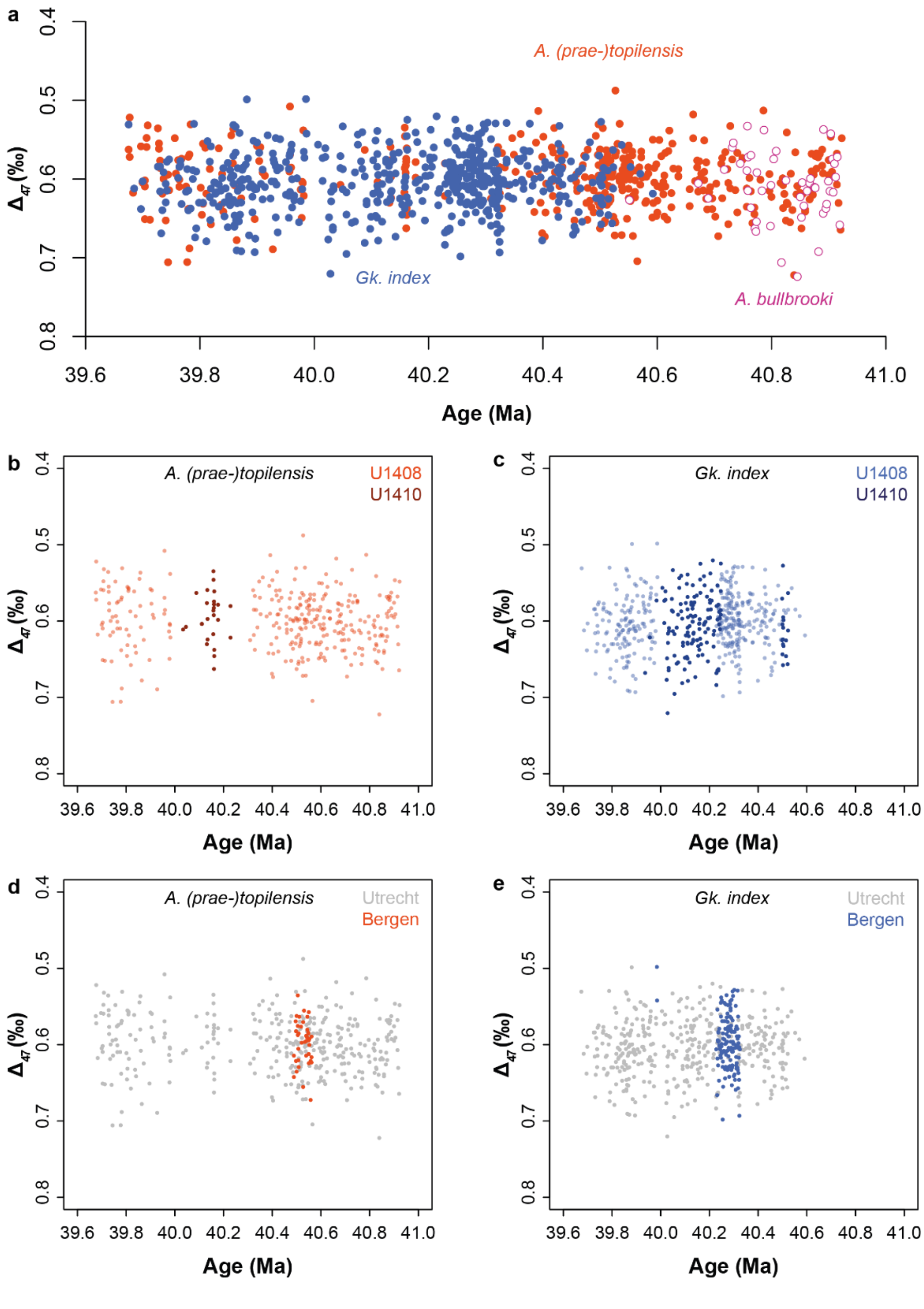

Fig. S5: Overview of all foraminiferal Δ47 data.

a, Δ47 data for *A. (prae-)topilensis* (in red), *A. bullbrooki* (in purple) and *Gk. index* (in blue), plotted against age following our revised composite age model for U1408 and U1410. **b–c,** Δ47 data for individual species groups *A. (prae-)topilensis* and *Gk. index* for Site U1408 (in light shade) and Site U1410 (in dark shade). **d–e,** Δ47 data for individual species groups *A. (prae-)topilensis* and *Gk. index* colored by the lab in which they were measured: Utrecht (in grey) and Bergen (in color).

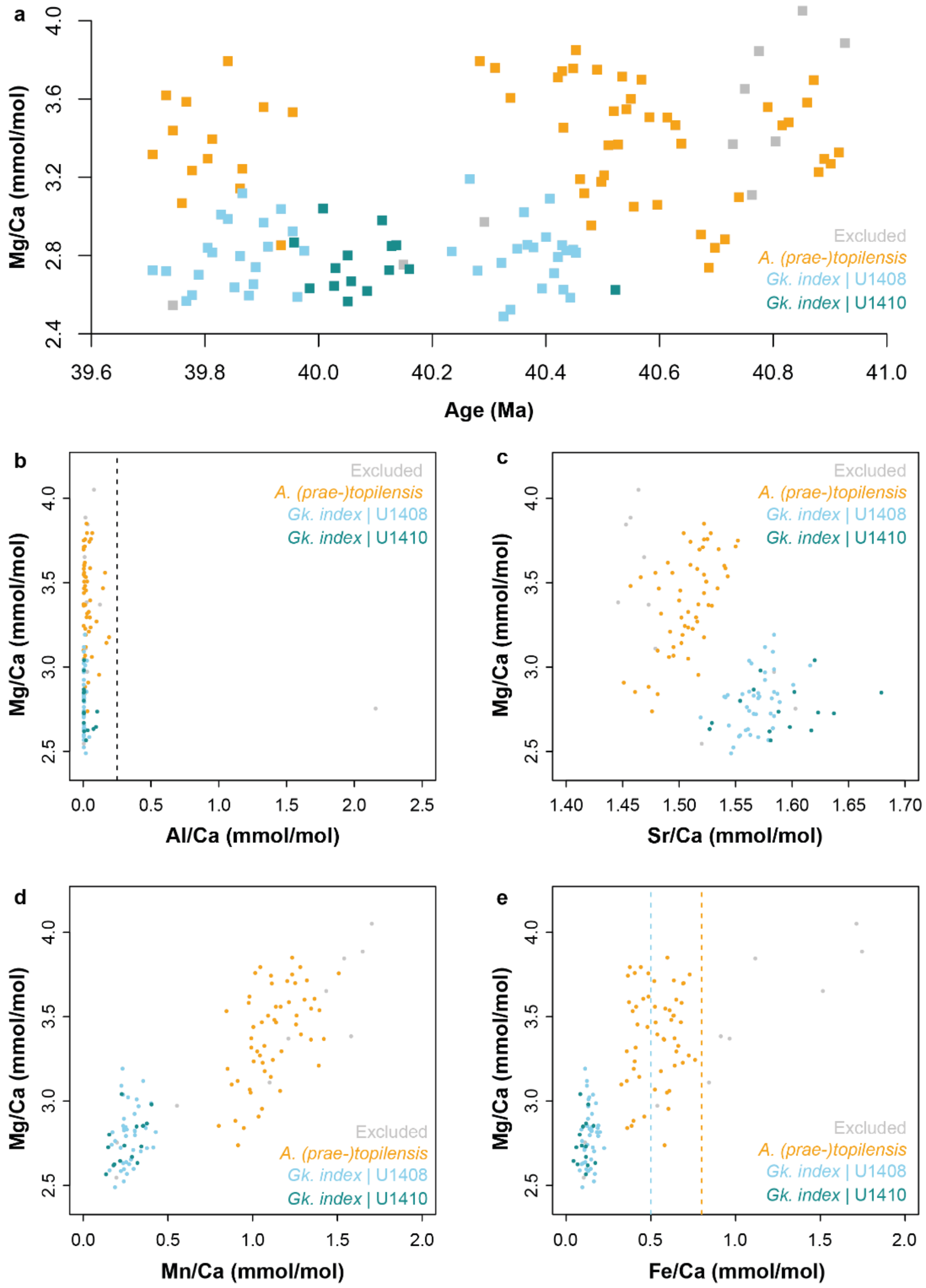

Fig. S6: Overview of all foraminiferal Mg/Ca data before and after trace element screening. **a,** Mg/Ca records for *A. (prae-)topilensis* (in orange) and *Gk. index* (in light blue), plotted against age following our revised composite age model for U1408 and U1410. b**,** Mg/Ca versus Al/Ca for *A. (prae-)topilensis* from Site U1408 (in orange), *Gk. index* from Site U1408 (in lightblue) and *Gk. index* from Site U1410 (in greenish blue). **c,** Mg/Ca versus Sr/Ca. **d,** Mg/Ca versus Mn/Ca. **e,** Mg/Ca versus Fe/Ca. All concentrations are in mmol/mol. with Measurements excluded on the basis of the criteria described in the Methods are shown in grey and respective cutoff values as dashed lines.

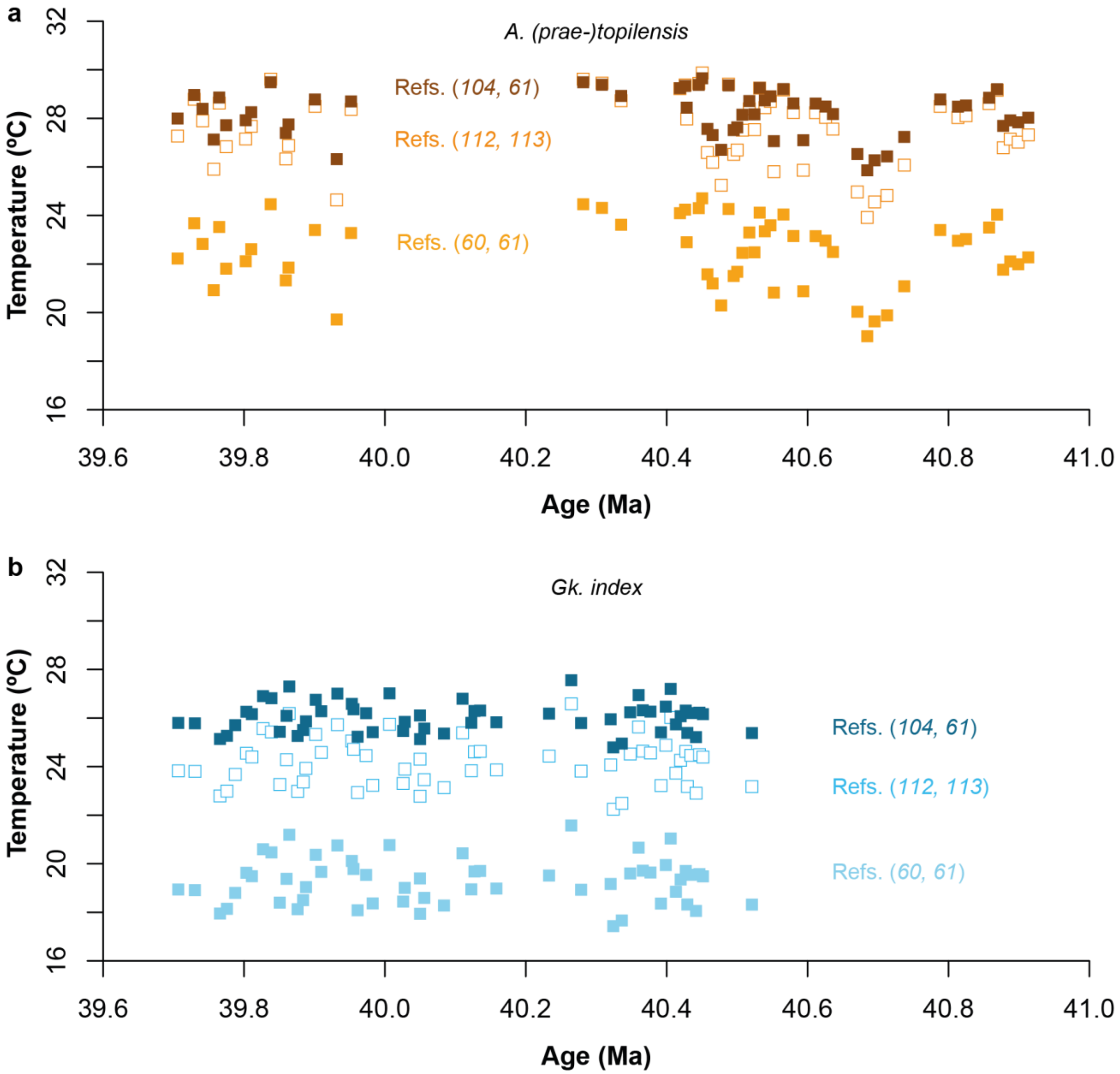

Fig. S7: Sensitivity of Mg/Ca-based temperature reconstructions to different Mg/Ca-temperature relationships.

a, Temperatures for *A. (prae-)topilensis* and **b,** *Gk. index* plotted against age following our revised composite age model for U1408 and U1410. Temperatures are calculated with the Mg/Ca-temperature relationships of (*60*) with modifications based on (*61*) (light orange/blue, closed symbols), (*112, 113*) (darker orange/blue, open symbols), or (*104*) with modifications based on (*61*) (darkest orange/blue, closed symbols). Mg/Ca-temperature relationships based on pH = 7.8, Mg/Ca_{sw} = 2.25 mmol/mol and S = 34 psu. See Methods for further details.

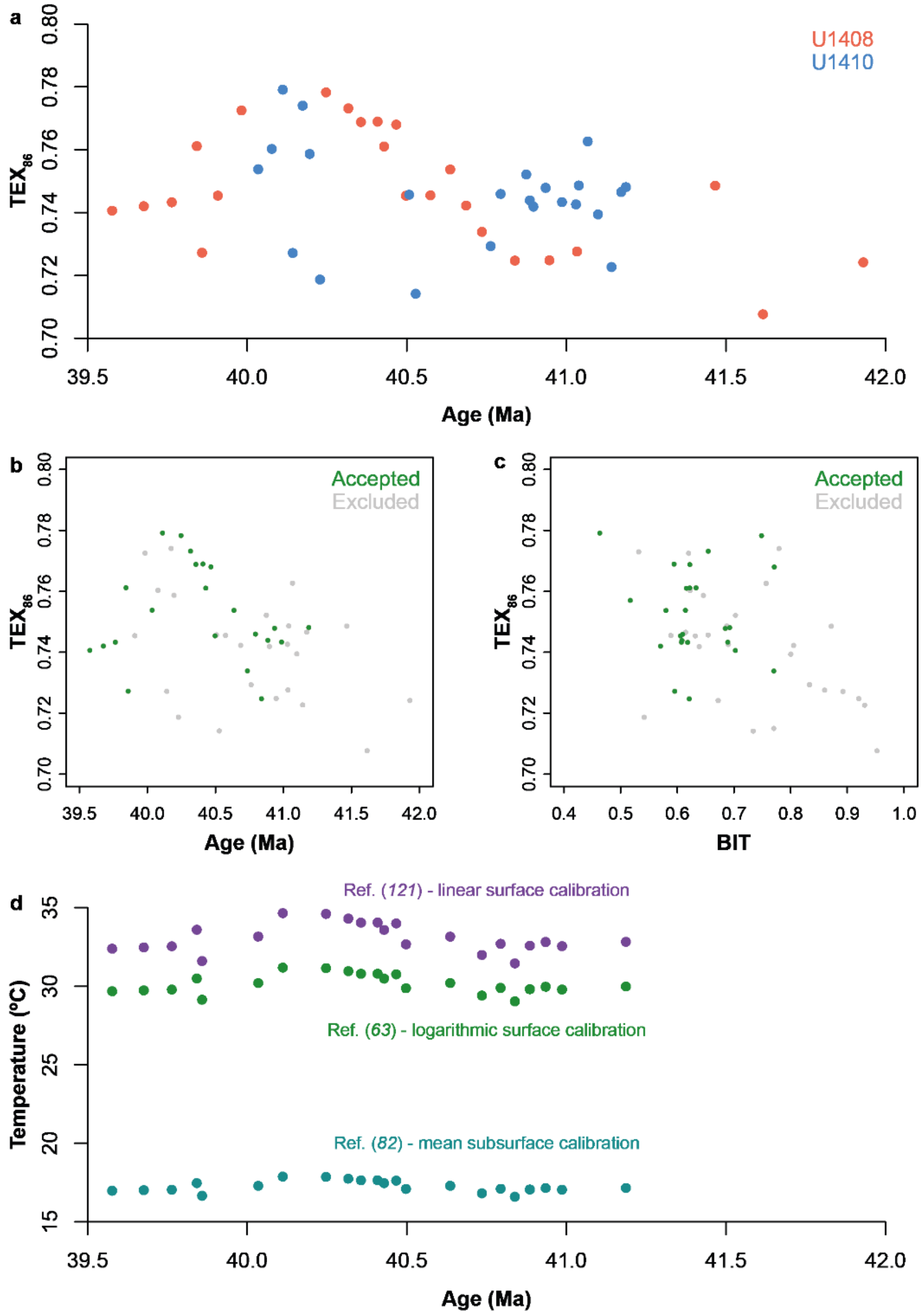

Fig. S8: Overview of all TEX_{86} data before and after GDGT distribution screening and sensitivity to different TEX_{86}-temperature relationships.

a, TEX_{86} plotted against age following our revised composite age model for Site U1408 (in red) and Site U1410 (in blue). **b,** Residual TEX_{86} data based on the evaluation of GDGT distributions as described in the Methods and visualized in Supplementary Figures 9–10, with accepted values in green and excluded values in grey. **c,** TEX_{86}-BIT relationship in the residual TEX_{86} data. Despite the very high absolute BIT values, there is no observable dependency between TEX_{86} and BIT. **d,** TEX_{86}-based temperatures calculated using the logarithmic sea surface calibration of (*63*) (in green), the linear sea surface calibration of (*121*) and the mean subsurface calibration of (*82*).

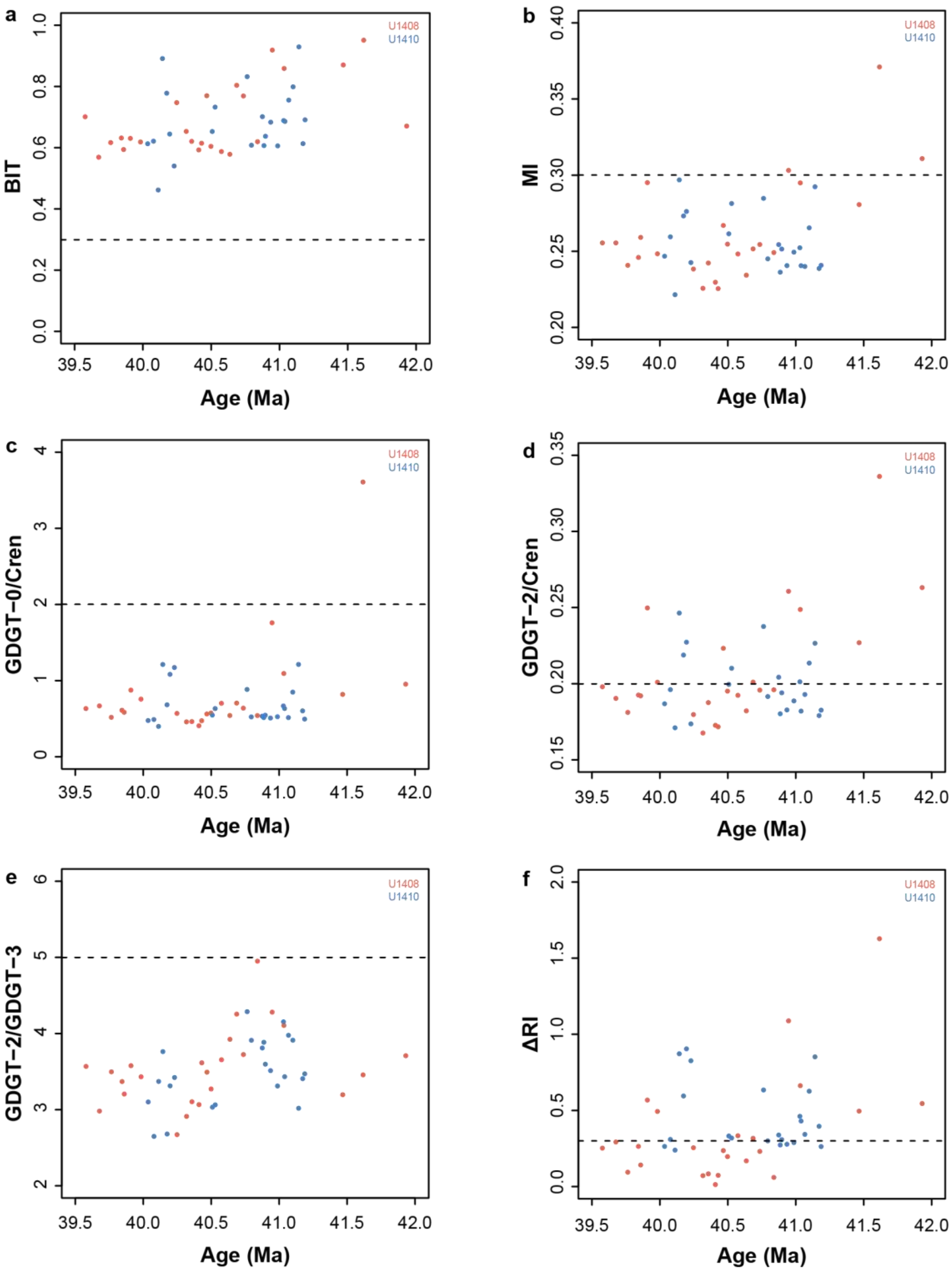

Fig. S9: Evaluation of GDGT distributions.

a, Branched versus Isoprenoid Tetraether (BIT) Index, **b,** Methane Index (MI), **c,** GDGT-0/crenarchaeol, **d,** GDGT-2/crenarchaeol, **e,** GDGT-2/GDGT-3 and **f,** ΔRing Index (ΔRI) plotted against age following our revised composite age model for Site U1408 (in red) and U1410 (in blue), with respective cutoff values shown as dashed lines.

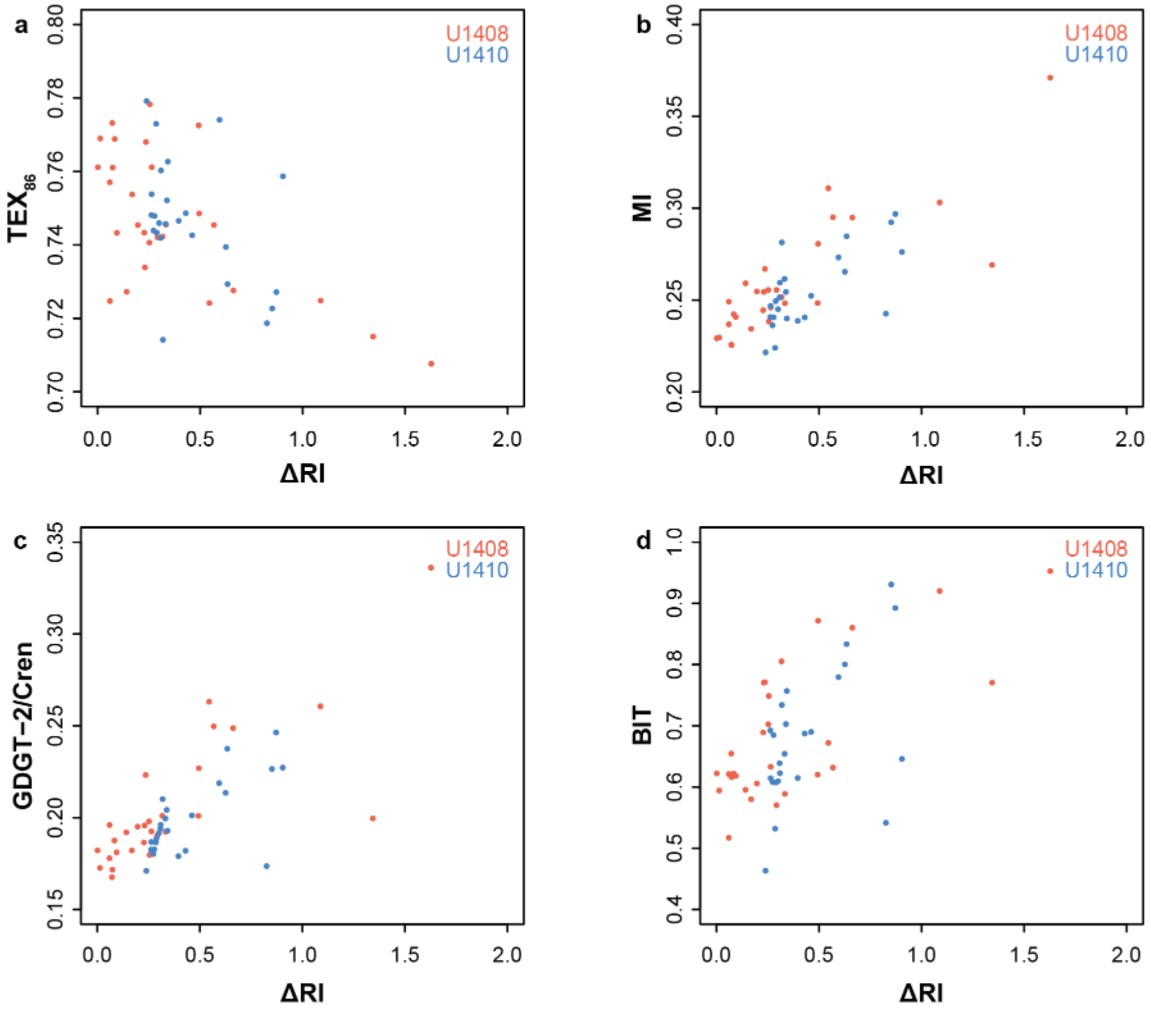

Fig. S10: TEX$_{86}$ and GDGT distributions versus ΔRI.

a-d, TEX$_{86}$, MI, GDGT-2/crenarchaeol and BIT plotted against ΔRI for Site U1408 (in red) and Site U1410 (in blue).

Table S1: Age model for Sites U1408 and U1410.

Depths correspond to the revised composite stratigraphy on the CCSF-X scale as presented in Figure 5 of (*41*).

**Depth (m CCSF-X)**	**Age (Ma)**
49.81	39.47
63.49	39.93
67.30	40.09
70.32	40.18
76.90	40.40
84.90	40.56
93.03	40.80
106.00	41.11
112.90	41.32
123.20	41.70
133.61	42.17
160.61	42.81
160.62	43.05
190.21	43.68
190.21	44.27
214.55	44.82
214.55	45.12
221.11	45.25
221.12	45.54
249.61	46.06

Supplementary Data (separate file): Excel spreadsheets containing the raw data presented in this study.

Data S1: Overview of all raw Δ_{47}, $\delta^{18}O_c$ and $\delta^{13}C_c$ data.
Data S2: Overview of clustered Δ_{47}, T, $\delta^{18}O_c$ and $\delta^{18}O_{sw}$ data.
Data S3: Overview of trace element and Mg/Ca data.
Data S4: Overview of $U^{K'}_{37}$, GDGT and TEX_{86} data.

REFERENCES

1. J. E. Tierney, C. J. Poulsen, I. P. Montañez, T. Bhattacharya, R. Feng, H. L. Ford, B. Hönisch, G. N. Inglis, S. V. Petersen, N. Sagoo, C. R. Tabor, K. Thirumalai, J. Zhu, N. J. Burls, G. L. Foster, Y. Goddéris, B. T. Huber, L. C. Ivany, S. K. Turner, D. J. Lunt, J. C. McElwain, B. J. W. Mills, B. L. Otto-Bliesner, A. Ridgwell, Y. G. Zhang, Past climates inform our future. *Science* **370**, aay3701 (2020).

2. E. Anagnostou, E. H. John, T. L. Babila, P. F. Sexton, A. Ridgwell, D. J. Lunt, P. N. Pearson, T. B. Chalk, R. D. Pancost, G. L. Foster, Proxy evidence for state-dependence of climate sensitivity in the Eocene greenhouse. *Nat. Commun.* **11**, 4436 (2020).

3. M. J. Cramwinckel, M. Huber, I. J. Kocken, C. Agnini, P. K. Bijl, S. M. Bohaty, J. Frieling, A. Goldner, F. J. Hilgen, E. L. Kip, F. Peterse, R. van der Ploeg, U. Röhl, S. Schouten, A. Sluijs, Synchronous tropical and polar temperature evolution in the Eocene. *Nature* **559**, 382–386 (2018).

4. C. J. Hollis, T. D. Jones, E. Anagnostou, P. K. Bijl, M. J. Cramwinckel, Y. Cui, G. R. Dickens, K. M. Edgar, Y. Eley, D. Evans, G. L. Foster, J. Frieling, G. N. Inglis, E. M. Kennedy, R. Kozdon, V. Lauretano, C. H. Lear, K. Littler, L. Lourens, A. Nele Meckler, B. David A. Naafs, H. Pälike, R. D. Pancost, P. N. Pearson, U. Röhl, D. L. Royer, U. Salzmann, B. A. Schubert, H. Seebeck, A. Sluijs, R. P. Speijer, P. Stassen, J. Tierney, A. Tripati, B. Wade, T. Westerhold, C. Witkowski, J. C. Zachos, Y. G. Zhang, M. Huber, D. J. Lunt, The DeepMIP contribution to PMIP4: Methodologies for selection, compilation and analysis of latest Paleocene and early Eocene climate proxy data, incorporating version 0.1 of the DeepMIP database. *Geosci. Model Dev.* **12**, 3149–3206 (2019).

5. S. Kirtland Turner, P. F. Sexton, C. D. Charles, R. D. Norris, Persistence of carbon release events through the peak of early Eocene global warmth. *Nat. Geosci.* **7**, 748–751 (2014).

6. J. C. Zachos, M. W. Wara, S. M. Bohaty, M. L. Delaney, M. R. Petrizzo, A. Brill, T. J. Bralower, I. Premoli-Silva, A transient rise in tropical sea surface temperature during the Paleocene-Eocene Thermal Maximum. *Science* **302**, 1551–1554 (2003).

7. M. Pagani, N. Pedentchouk, M. Huber, A. Sluijs, S. Schouten, H. Brinkhuis, J. S. Sinninghe Damsté, G. R. Dickens, Arctic hydrology during global warming at the Palaeocene/Eocene thermal maximum. *Nature* **442**, 671–675 (2006).

8. J. Frieling, H. Gebhardt, M. Huber, O. A. Adekeye, S. O. Akande, G.-J. Reichart, J. J. Middelburg, S. Schouten, A. Sluijs, Extreme warmth and heat-stressed plankton in the tropics during the Paleocene-Eocene Thermal Maximum. *Sci. Adv.* **3**, e1600891 (2017).

9. S. M. Bohaty, J. C. Zachos, Significant Southern Ocean warming event in the late middle Eocene. *Geology* **31**, 1017–1020 (2003).

10. S. M. Bohaty, J. C. Zachos, F. Florindo, M. L. Delaney, Coupled greenhouse warming and deep-sea acidification in the middle Eocene. *Paleoceanography* **24**, PA2207 (2009).

11. A. Sluijs, R. E. Zeebe, P. K. Bijl, S. M. Bohaty, A middle Eocene carbon cycle conundrum. *Nat. Geosci.* **6**, 429–434 (2013).

12. M. J. Henehan, K. M. Edgar, G. L. Foster, D. E. Penman, P. M. Hull, R. Greenop, E. Anagnostou, P. N. Pearson, Revisiting the Middle Eocene Climatic Optimum "Carbon Cycle Conundrum" with new estimates of atmospheric pCO_2 from boron isotopes. *Paleoceanogr. Paleoclimatol.* **35**, e2019PA003713 (2020).

13. I. M. Held, B. J. Soden, Robust responses of the hydrological cycle to global warming. *J. Climate* **19**, 5686–5699 (2006).

14. N. J. Burls, A. V. Fedorov, Wetter subtropics in a warmer world: Contrasting past and future hydrological cycles. *Proc. Natl. Acad. Sci. U.S.A.* **114**, 12888–12893 (2017).

15. A. Sluijs, S. Schouten, T. H. Donders, P. L. Schoon, U. Röhl, G.-J. Reichart, F. Sangiorgi, J.-H. Kim, J. S. Sinninghe Damsté, H. Brinkhuis, Warm and wet conditions in the arctic region during Eocene Thermal Maximum 2. *Nat. Geosci.* **2**, 777–780 (2009).

16. J. C. Zachos, S. Schouten, S. Bohaty, T. Quattlebaum, A. Sluijs, H. Brinkhuis, S. J. Gibbs, T. J. Bralower, Extreme warming of mid-latitude coastal ocean during the Paleocene-Eocene

Thermal Maximum: Inferences from TEX86 and isotope data. *Geology* **34**, 737–740 (2006).

17. A. K. Tripati, H. Elderfield, Abrupt hydrographic changes in the equatorial Pacific and subtropical Atlantic from foraminiferal Mg/Ca indicate greenhouse origin for the thermal maximum at the Paleocene-Eocene Boundary. *Geochem. Geophys. Geosyst.* **5**, 1–11 (2004).

18. D. T. Harper, R. Zeebe, B. Hönisch, C. D. Schrader, L. J. Lourens, J. C. Zachos, Subtropical sea-surface warming and increased salinity during Eocene Thermal Maximum 2. *Geology* **46**, 187–190 (2018).

19. P. K. Bijl, A. J. P. Houben, S. Schouten, S. M. Bohaty, A. Sluijs, G.-J. Reichart, J. S. Sinninghe Damsté, H. Brinkhuis, Transient Middle Eocene atmospheric CO_2 and temperature variations. *Science* **330**, 819–821 (2010).

20. F. Boscolo Galazzo, L. Giusberti, V. Luciani, E. Thomas, Paleoenvironmental changes during the Middle Eocene Climatic Optimum (MECO) and its aftermath: The benthic foraminiferal record from the Alano section (NE Italy). *Palaeogeogr. Palaeoclimatol. Palaeoecol.* **378**, 22–35 (2013).

21. F. Boscolo Galazzo, E. Thomas, L. Giusberti, Benthic foraminiferal response to the Middle Eocene Climatic Optimum (MECO) in the South-Eastern Atlantic (ODP Site 1263). *Palaeogeogr. Palaeoclimatol. Palaeoecol.* **417**, 432–444 (2015).

22. R. van der Ploeg, D. Selby, M. J. Cramwinckel, Y. Li, S. M. Bohaty, J. J. Middelburg, A. Sluijs, Middle Eocene greenhouse warming facilitated by diminished weathering feedback. *Nat. Commun.* **9**, 2877 (2018).

23. A. van der Boon, K. F. Kuiper, R. van der Ploeg, M. J. Cramwinckel, M. Honarmand, A. Sluijs, W. Krijgsman, Exploring a link between the Middle Eocene Climatic Optimum and Neotethys continental arc flare-up. *Clim. Past* **17**, 229–239 (2021).

24. M. J. Cramwinckel, L. Woelders, E. P. Huurdeman, F. Peterse, S. J. Gallagher, J. Pross, C. E. Burgess, G. J. Reichart, A. Sluijs, P. K. Bijl, Surface-circulation change in the southwest Pacific Ocean across the Middle Eocene Climatic Optimum: Inferences from dinoflagellate

cysts and biomarker paleothermometry. *Clim. Past.* **16**, 1667–1689 (2020).

25. L. Jovane, F. Florindo, R. Coccioni, J. Dinarès-Turell, A. Marsili, S. Monechi, A. P. Roberts, M. Sprovieri, The middle Eocene climatic optimum event in the Contessa Highway section, Umbrian Apennines, Italy, *Geol. Soc. Am. Bull.* **119**, 413–427 (2007).

26. D. J. A. Spofforth, C. Agnini, H. Pälike, D. Rio, E. Fornaciari, L. Giusberti, V. Luciani, L. Lanci, G. Muttoni, Organic carbon burial following the Middle Eocene Climatic Optimum in the central western Tethys. *Paleoceanography*. **25**, PA3210 (2010).

27. M. Giorgioni, L. Jovane, E. S. Rego, D. Rodelli, F. Frontalini, R. Coccioni, R. Catanzariti, E. Özcan, Carbon cycle instability and orbital forcing during the Middle Eocene Climatic Optimum. *Sci. Rep.* **9**, 9357 (2019).

28. M. J. Cramwinckel, R. van der Ploeg, N. A. G. M. van Helmond, N. Waarlo, C. Agnini, P. K. Bijl, A. van der Boon, H. Brinkhuis, J. Frieling, W. Krijgsman, T. A. Mather, J. J. Middelburg, F. Peterse, C. P. Slomp, A. Sluijs, Deoxygenation and organic carbon sequestration in the Tethyan realm associated with the Middle Eocene Climatic Optimum. *GSA Bull.* 10.1130/B36280.1 (2022).

29. K. M. Edgar, P. A. Wilson, P. F. Sexton, S. J. Gibbs, A. P. Roberts, R. D. Norris, New biostratigraphic, magnetostratigraphic and isotopic insights into the Middle Eocene Climatic Optimum in low latitudes. *Palaeogeogr. Palaeoclimatol. Palaeoecol.* **297**, 670–682 (2010).

30. F. Boscolo Galazzo, E. Thomas, M. Pagani, C. Warren, V. Luciani, L. Giusberti, The Middle Eocene Climatic Optimum: (MECO): A multiproxy record of paleoceanographic changes in the southeast Atlantic (ODP Site 1263, Walvis Ridge). *Paleoceanography* **29**, 1143–1161 (2014).

31. M. J. Cramwinckel, H. K. Coxall, K. K. Śliwińska, M. Polling, D. T. Harper, P. K. Bijl, H. Brinkhuis, J. S. Eldrett, A. J. P. Houben, F. Peterse, S. Schouten, G. Reichart, J. C. Zachos, A. Sluijs, A warm, stratified, and restricted Labrador Sea across the middle Eocene and its Climatic Optimum. *Paleoceanogr Paleoclimatol.* ,**35** e2020PA003932 (2020).

32. J. Arimoto, H. Nishi, A. Kuroyanagi, R. Takashima, H. Matsui, M. Ikehara, Changes in upper ocean hydrography and productivity across the Middle Eocene Climatic Optimum: Local insights and global implications from the Northwest Atlantic. *Glob. Planet. Change* **193**, 103258 (2020).

33. M. J. Cramwinckel, R. van der Ploeg, P. K. Bijl, F. Peterse, S. M. Bohaty, U. Röhl, S. Schouten, J. J. Middelburg, A. Sluijs, Harmful algae and export production collapse in the equatorial Atlantic during the zenith of Middle Eocene Climatic Optimum warmth. *Geology* **47**, 247–250 (2019).

34. K. M. Edgar, S. M. Bohaty, H. K. Coxall, P. R. Bown, S. J. Batenburg, C. H. Lear, P. N. Pearson, New composite bio- And isotope stratigraphies spanning the Middle Eocene Climatic Optimum at tropical ODP Site 865 in the Pacific Ocean. *J. Micropalaeontol.* **39**, 117–138 (2020).

35. J. Tindall, R. Flecker, P. Valdes, D. N. Schmidt, P. Markwick, J. Harris, Modelling the oxygen isotope distribution of ancient seawater using a coupled ocean-atmosphere GCM: Implications for reconstructing early Eocene climate. *Earth Planet. Sci. Lett.* **292**, 265–273 (2010).

36. C. D. Roberts, A. N. Legrande, A. K. Tripati, Sensitivity of seawater oxygen isotopes to climatic and tectonic boundary conditions in an early Paleogene simulation with GISS ModelE-R. *Paleoceanography* **26**, 1–16 (2011).

37. J. Zhu, C. J. Poulsen, B. L. Otto-Bliesner, Z. Liu, E. C. Brady, D. C. Noone, Simulation of early Eocene water isotopes using an Earth system model and its implication for past climate reconstruction. *Earth Planet. Sci. Lett.* **537**, 116164 (2020).

38. D. J. J. van Hinsbergen, L. V. de Groot, S. J. van Schaik, W. Spakman, P. K. Bijl, A. Sluijs, C. G. Langereis, H. Brinkhuis, A paleolatitude calculator for paleoclimate studies. *PLOS ONE* **10**, e0126946 (2015).

39. R. D. Norris, P. A. Wilson, P. Blum, A. Fehr, C. Agnini, A. Bornemann, S. Boulila, P. R.

Bown, C. Cournede, O. Friedrich, A. K. Ghosh, C. J. Hollis, P. M. Hull, K. Jo, C. K. Junium, M. Kaneko, D. Liebrand, P. C. Lippert, Z. Liu, H. Matsui, K. Moriya, H. Nishi, B. N. Opdyke, D. Penman, B. Romans, H. D. Scher, P. Sexton, H. Takagi, S. K. Turner, J. H. Whiteside, T. Yamaguchi, Y. Yamamoto, in *Proceedings of the Integrated Ocean Drilling Program* (2014), vol. 342; http://publications.iodp.org/proceedings/342/101/101_.htm.

40. P. R. Boyle, B. W. Romans, B. E. Tucholke, R. D. Norris, S. A. Swift, P. F. Sexton, Cenozoic North Atlantic deep circulation history recorded in contourite drifts, offshore Newfoundland, Canada, *Mar. Geol.* **385**, 185–203 (2017).

41. D. De Vleeschouwer, D. E. Penman, S. D'haenens, F. Wu, T. Westerhold, M. Vahlenkamp, C. Cappelli, C. Agnini, W. E. C. Kordesch, D. King, R. Van der Ploeg, H. Pälike, S. Kirtland Turner, P. A. Wilson, R. D. Norris, J. C. Zachos, S. M. Bohaty, P. M. Hull, North Atlantic drift sediments constrain Eocene tidal dissipation and the evolution of the Earth-Moon system. EarthArXiv:X53H1W [**Preprint**]. 15 September 2022. https://doi.org/10.31223/X53H1W.

42. T. J. Leutert, A. Auderset, A. Martínez-García, S. Modestou, A. N. Meckler, Coupled Southern Ocean cooling and Antarctic ice sheet expansion during the middle Miocene. *Nat. Geosci.* **13**, 634–639 (2020).

43. A. N. Meckler, P. F. Sexton, A. M. Piasecki, T. J. Leutert, J. Marquardt, M. Ziegler, T. Agterhuis, L. J. Lourens, J. W. B. Rae, J. Barnet, A. Tripati, S. M. Bernasconi, Cenozoic evolution of deep ocean temperature from clumped isotope thermometry. *Science* **377**, 86–90 (2022).

44. J. M. Eiler, Paleoclimate reconstruction using carbonate clumped isotope thermometry. *Quat. Sci. Rev.* **30**, 3575–3588 (2011).

45. N. Meinicke, S. L. Ho, B. Hannisdal, D. Nürnberg, A. Tripati, R. Schiebel, A. N. Meckler, A robust calibration of the clumped isotopes to temperature relationship for foraminifers. *Geochim. Cosmochim. Acta* **270**, 160–183 (2020).

46. S. M. Bernasconi, M. Daëron, K. D. Bergmann, M. Bonifacie, A. N. Meckler, H. P. Affek, N. Anderson, D. Bajnai, E. Barkan, E. Beverly, D. Blamart, L. Burgener, D. Calmels, C. Chaduteau, M. Clog, B. Davidheiser-Kroll, A. Davies, F. Dux, J. Eiler, B. Elliott, A. C. Fetrow, J. Fiebig, S. Goldberg, M. Hermoso, K. W. Huntington, E. Hyland, M. Ingalls, M. Jaggi, C. M. John, A. B. Jost, S. Katz, J. Kelson, T. Kluge, I. J. Kocken, A. Laskar, T. J. Leutert, D. Liang, J. Lucarelli, T. J. Mackey, X. Mangenot, N. Meinicke, S. E. Modestou, I. A. Müller, S. Murray, A. Neary, N. Packard, B. H. Passey, E. Pelletier, S. Petersen, A. Piasecki, A. Schauer, K. E. Snell, P. K. Swart, A. Tripati, D. Upadhyay, T. Vennemann, I. Winkelstern, D. Yarian, N. Yoshida, N. Zhang, M. Ziegler, InterCarb: A community effort to improve interlaboratory standardization of the carbonate clumped isotope thermometer using carbonate standards. *Geochem. Geophys. Geosyst.* **22**, e2020GC009588 (2021).

47. T. J. Leutert, P. F. Sexton, A. Tripati, A. Piasecki, S. L. Ho, A. N. Meckler, Sensitivity of clumped isotope temperatures in fossil benthic and planktic foraminifera to diagenetic alteration. *Geochim. Cosmochim. Acta* **257**, 354–372 (2019).

48. R. D. Norris, P. A. Wilson, P. Blum, A. Fehr, C. Agnini, A. Bornemann, S. Boulila, P. R. Bown, C. Cournede, O. Friedrich, A. K. Ghosh, C. J. Hollis, P. M. Hull, K. Jo, C. K. Junium, M. Kaneko, D. Liebrand, P. C. Lippert, Z. Liu, H. Matsui, K. Moriya, H. Nishi, B. N. Opdyke, D. Penman, B. Romans, H. D. Scher, P. Sexton, H. Takagi, S. K. Turner, J. H. Whiteside, T. Yamaguchi, Y. Yamamoto, in *Proceedings of the Integrated Ocean Drilling Program* (2014), vol. 342, pp. 1–91; http://publications.iodp.org/proceedings/342/109/109_.htm.

49. R. D. Norris, P. A. Wilson, P. Blum, A. Fehr, C. Agnini, A. Bornemann, S. Boulila, P. R. Bown, C. Cournede, O. Friedrich, A. K. Ghosh, C. J. Hollis, P. M. Hull, K. Jo, C. K. Junium, M. Kaneko, D. Liebrand, P. C. Lippert, Z. Liu, H. Matsui, K. Moriya, H. Nishi, B. N. Opdyke, D. Penman, B. Romans, H. D. Scher, P. Sexton, H. Takagi, S. K. Turner, J. H. Whiteside, T. Yamaguchi, Y. Yamamoto, in *Proceedings of the Integrated Ocean Drilling Program* (2014), vol. 342, pp. 1–87; http://publications.iodp.org/proceedings/342/111/111_.htm.

50. P. N. Pearson, P. W. Ditchfield, J. Singano, K. G. Harcourt-Brown, C. J. Nicholas, R. K. Olsson, N. J. Shackleton, M. A. Hall, Warm tropical sea surface temperatures in the Late Cretaceous and Eocene epochs. *Nature* **414**, 470 (2001).

51. P. F. Sexton, P. A. Wilson, P. N. Pearson, Microstructural and geochemical perspectives on planktic foraminiferal preservation: "Glassy" versus "frosty". *Geochem. Geophys. Geosystems* **7**, 10.1029/2006GC001291 (2006).

52. I. Premoli-Silva, B. S. Wade, P. N. Pearson, Taxonomy, biostratigraphy, and phylogeny of *Globigerinatheka* and *Orbulinoides*, in *Atlas of Eocene Planktonic Foraminifera* (Cushman Foundation Special Publication, 2006), vol. 41, pp. 169–212.

53. P. F. Sexton, P. A. Wilson, P. N. Pearson, Palaeoecology of late middle Eocene planktic foraminifera and evolutionary implications. *Mar. Micropaleontol.* **60**, 1–16 (2006).

54. S. Boulila, M. Vahlenkamp, D. De Vleeschouwer, J. Laskar, Y. Yamamoto, H. Pälike, S. Kirtland Turner, P. F. Sexton, T. Westerhold, U. Röhl, Towards a robust and consistent middle Eocene astronomical timescale. *Earth Planet. Sci. Lett.* **486**, 94–107 (2018).

55. M. Vahlenkamp, I. Niezgodzki, D. De Vleeschouwer, T. Bickert, D. Harper, S. Kirtland Turner, G. Lohmann, P. Sexton, J. Zachos, H. Pälike, Astronomically paced changes in deep-water circulation in the western North Atlantic during the middle Eocene. *Earth Planet. Sci. Lett.* **484**, 329–340 (2018).

56. W. Si, M.-P. Aubry, Vital effects and ecologic adaptation of photosymbiont-bearing planktonic foraminifera during the Paleocene-Eocene Thermal Maximum, implications for paleoclimate. *Paleoceanogr. Paleoclimatol.* 112–125 (2018).

57. V. Luciani, G. R. Dickens, J. Backman, E. Fornaciari, L. Giusberti, C. Agnini, R. D'Onofrio, Major perturbations in the global carbon cycle and photosymbiont-bearing planktic foraminifera during the early Eocene. *Clim. Past* **12**, 981–1007 (2016).

58. M. R. Petrizzo, G. Leoni, R. P. Speijer, B. De Bernardi, F. Felletti, Dissolution susceptibility of some paleogene planktonic foraminifera from ODP site 1209 (Shatsky rise, Pacific

Ocean). *J. Foraminifer. Res.* **38**, 357–371 (2008).

59. K. M. Edgar, S. M. Bohaty, S. J. Gibbs, P. F. Sexton, R. D. Norris, P. A. Wilson, Symbiont 'bleaching' in planktic foraminifera during the Middle Eocene Climatic Optimum. *Geology* **41**, 15–18 (2013).

60. W. R. Gray, S. Weldeab, D. W. Lea, Y. Rosenthal, N. Gruber, B. Donner, G. Fischer, The effects of temperature, salinity, and the carbonate system on Mg/Ca in *Globigerinoides ruber* (white): A global sediment trap calibration. *Earth Planet. Sci. Lett.* **482**, 607–620 (2018).

61. D. Evans, W. Müller, Deep time foraminifera Mg/Ca paleothermometry: Nonlinear correction for secular change in seawater Mg/Ca. *Paleoceanography* **27**, 4205 (2012).

62. J. W. H. Weijers, S. Schouten, O. C. Spaargaren, J. S. Sinninghe Damsté, Occurrence and distribution of tetraether membrane lipids in soils: Implications for the use of the TEX_{86} proxy and the BIT index. *Org. Geochem.* **37**, 1680–1693 (2006).

63. J.-H. Kim, J. van der Meer, S. Schouten, P. Helmke, V. Willmott, F. Sangiorgi, N. Koç, E. C. Hopmans, J. S. S. Damsté, New indices and calibrations derived from the distribution of crenarchaeal isoprenoid tetraether lipids: Implications for past sea surface temperature reconstructions. *Geochim. Cosmochim. Acta* **74**, 4639–4654 (2010).

64. J. E. Tierney, M. P. Tingley, A Bayesian, spatially-varying calibration model for the TEX_{86} proxy. *Geochim. Cosmochim. Acta* **127**, 83–106 (2014).

65. J. E. Tierney, M. P. Tingley, BAYSPLINE: A new calibration for the alkenone paleothermometer. *Paleoceanogr. Paleoclimatol.* **33**, 281–301 (2018).

66. Z. Liu, Y. He, Y. Jiang, H. Wang, W. Liu, S. M. Bohaty, P. A. Wilson, Transient temperature asymmetry between hemispheres in the Palaeogene Atlantic Ocean. *Nat. Geosci.* **11**, 656–660 (2018).

67. J. C. Zachos, L. D. Stott, K. C. Lohmann, Evolution of early Cenozoic marine temperatures. *Paleoceanogr. Paleoclimatol.* **9**, 353–387 (1994).

68. P. N. Pearson, Oxygen isotopes in foraminifera: Overview and historical review. *Paleontol. Soc. Pap.* **18**, 1–38 (2012).

69. S. Krishnan, M. Pagani, M. Huber, A. Sluijs, High latitude hydrological changes during the Eocene Thermal Maximum 2. *Earth Planet. Sci. Lett.* **404**, 167–177 (2014).

70. R. E. Zeebe, Seawater pH and isotopic paleotemperatures of Cretaceous oceans. *Palaeogeogr. Palaeoclimatol. Palaeoecol.* **170**, 49–57 (2001).

71. G. A. Schmidt, Forward modeling of carbonate proxy data from planktonic foraminifera using oxygen isotope tracers in a global ocean model. *Paleoceanogr. Paleoclimatol.* **14**, 482–497 (1999).

72. S. J. Levang, R. W. Schmitt, Centennial changes of the global water cycle in CMIP5 models. *J. Clim.* **28**, 6489–6502 (2015).

73. L. Handley, A. O'Halloran, P. N. Pearson, E. Hawkins, C. J. Nicholas, S. Schouten, I. K. McMillan, R. D. Pancost, Changes in the hydrological cycle in tropical East Africa during the Paleocene–Eocene Thermal Maximum. *Palaeogeogr. Palaeoclimatol. Palaeoecol.* **329–330**, 10–21 (2012).

74. E. N. Speelman, J. O. Sewall, D. Noone, M. Huber, A. von der Heydt, J. S. Damsté, G. J. Reichart, Modeling the influence of a reduced equator-to-pole sea surface temperature gradient on the distribution of water isotopes in the Early/Middle Eocene. *Earth Planet. Sci. Lett.* **298**, 57–65 (2010).

75. A. Filippova, M. Kienast, M. Frank, R. R. Schneider, Alkenone paleothermometry in the North Atlantic: A review and synthesis of surface sediment data and calibrations. *Geochem. Geophys. Geosyst.* **17**, 1370–1382 (2016).

76. C. Wuchter, S. Schouten, S. G. Wakeham, J. S. S. Damsté, Archaeal tetraether membrane lipid fluxes in the northeastern Pacific and the Arabian Sea: Implications for TEX_{86} paleothermometry. *Paleoceanography* **21**, 1–9 (2006).

77. J. A. Yoder, C. R. McClain, G. C. Feldman, W. E. Esaias, Annual cycles of phytoplankton chlorophyll concentrations in the global ocean: A satellite view. *Global Biogeochem. Cycles* **7**, 181–193 (1993).

78. J. N. Richey, J. E. Tierney, GDGT and alkenone flux in the northern Gulf of Mexico: Implications for the TEX_{86} and $U^{K'}_{37}$ paleothermometers. *Paleoceanogr. Paleoclimatol.* **31**, 1547–1561 (2016).

79. N. Ohkouchi, T. I. Eglinton, L. D. Keigwin, J. M. Hayes, Spatial and temporal offsets between proxy records in a sediment drift. *Science* **298**, 1224–1227 (2002).

80. J.-H. Kim, X. Crosta, E. Michel, S. Schouten, J. Duprat, J. S. Sinninghe Damsté, Impact of lateral transport on organic proxies in the Southern Ocean. *Quat. Res.* **71**, 246–250 (2009).

81. P. D. Nooteboom, P. K. Bijl, E. van Sebille, A. S. von der Heydt, H. A. Dijkstra, Transport bias by ocean currents in sedimentary microplankton assemblages: Implications for paleoceanographic reconstructions. *Paleoceanogr. Paleoclimatol.* **34**, 1178–1194 (2019).

82. S. L. Ho, T. Laepple, Flat meridional temperature gradient in the early Eocene in the subsurface rather than surface ocean. *Nat. Geosci.* **9**, 606–610 (2016).

83. D. A. Stolper, J. M. Eiler, J. A. Higgins, Modeling the effects of diagenesis on carbonate clumped-isotope values in deep- and shallow-water settings. *Geochim. Cosmochim. Acta* **227**, 264–291 (2018).

84. B. H. Passey, G. A. Henkes, Carbonate clumped isotope bond reordering and geospeedometry. *Earth Planet. Sci. Lett.* **351–352**, 223–236 (2012).

85. M. Regenberg, A. Regenberg, D. Garbe-Schönberg, D. W. Lea, Global dissolution effects on planktonic foraminiferal Mg/Ca ratios controlled by the calcite-saturation state of bottom waters. *Paleoceanogr. Paleoclimatol.* **29**, 127–142 (2014).

86. S. F. M. Breitenbach, M. J. Mleneck-Vautravers, A.-L. Grauel, L. Lo, S. M. Bernasconi, I. A. Müller, J. Rolfe, F. Gázquez, M. Greaves, D. A. Hodell, Coupled Mg/Ca and clumped

isotope analyses of foraminifera provide consistent water temperatures. *Geochim. Cosmochim. Acta* **236**, 283–296 (2018).

87. P. N. Pearson, C. E. Burgess, Foraminifer test preservation and diagenesis: Comparison of high latitude Eocene sites. *Geol. Soc. London, Spec. Publ.* **303**, 59–72 (2008).

88. H. K. Coxall, C. E. Huck, M. Huber, C. H. Lear, A. Legarda-Lisarri, M. O'Regan, K. K. Sliwinska, T. van de Flierdt, A. M. de Boer, J. C. Zachos, J. Backman, Export of nutrient rich Northern component water preceded early Oligocene Antarctic glaciation. *Nat. Geosci.* **11**, 190–196 (2018).

89. S. Barker, M. Greaves, H. Elderfield, A study of cleaning procedures used for foraminiferal Mg/Ca paleothermometry. *Geochem. Geophys. Geosyst.* **4**, 8407 (2003).

90. T. W. Schmid, S. M. Bernasconi, An automated method for 'clumped-isotope' measurements on small carbonate samples. *Rapid Commun. Mass Spectrom.* **24**, 1955–1963 (2010).

91. B. Hu, J. Radke, H.-J. Schlüter, F. T. Heine, L. Zhou, S. M. Bernasconi, A modified procedure for gas-source isotope ratio mass spectrometry: The long-integration dual-inlet (LIDI) methodology and implications for clumped isotope measurements. *Rapid Commun. Mass Spectrom.* **28**, 1413–1425 (2014).

92. B. He, G. A. Olack, A. S. Colman, Pressure baseline correction and high-precision CO_2 clumped-isotope (Δ_{47}) measurements in bellows and micro-volume modes. *Rapid Commun. Mass Spectrom.* **26**, 2837–2853 (2012).

93. S. M. Bernasconi, I. A. Müller, K. D. Bergmann, S. F. M. Breitenbach, A. Fernandez, D. A. Hodell, M. Jaggi, A. N. Meckler, I. Millan, M. Ziegler, Reducing uncertainties in carbonate clumped isotope analysis through consistent carbonate-based standardization. *Geochem. Geophys. Geosyst.* **19**, 2895–2914 (2018).

94. R Core Team, R: A language and environment for statistical computing (R Foundation for Statistical Computing, Vienna, Austria, 2020).

95. C. M. John, D. Bowen, Community software for challenging isotope analysis: First applications of ‘Easotope’ to clumped isotopes. *Rapid Commun. Mass Spectrom.* **30**, 2285–2300 (2016).

96. N. Meinicke, M. A. Reimi, A. C. Ravelo, A. N. Meckler, Coupled Mg/Ca and clumped isotope measurements indicate lack of substantial mixed layer cooling in the Western Pacific warm pool during the last ∼5 million years. *Paleoceanogr. Paleoclimatol.* **36**, e2020PA004115 (2021).

97. L. Rodríguez-Sanz, S. M. Bernasconi, G. Marino, D. Heslop, I. A. Müller, A. Fernandez, K. M. Grant, E. J. Rohling, Penultimate deglacial warming across the Mediterranean Sea revealed by clumped isotopes in foraminifera. *Sci. Rep.* **7**, 16572 (2017).

98. M. Peral, M. Daëron, D. Blamart, F. Bassinot, F. Dewilde, N. Smialkowski, G. Isguder, J. Bonnin, F. Jorissen, C. Kissel, E. Michel, N. Vázquez Riveiros, C. Waelbroeck, Updated calibration of the clumped isotope thermometer in planktonic and benthic foraminifera. *Geochim. Cosmochim. Acta* **239**, 1–16 (2018).

99. S. T. Kim, J. R. O’Neil, Equilibrium and nonequilibrium oxygen isotope effects in synthetic carbonates. *Geochim. Cosmochim. Acta* **61**, 3461–3475 (1997).

100. B. E. Bemis, H. J. Spero, J. Bijma, D. W. Lea, Reevaluation of the oxygen isotopic composition of planktonic foraminifera: Experimental results and revised paleotemperature equations. *Paleoceanogr. Paleoclimatol.* **13**, 150–160 (1998).

101. H. G. Ostlund, *GEOSECS Atlantic, Pacific, and Indian Ocean Expeditions Vol 7* (1987).

102. J.-C. Duplessy, L. Labeyrie, A. Juillet-Leclerc, F. Maitre, J. Duprat, M. Sarnthein, Surface salinity reconstruction of the north-atlantic ocean during the last glacial maximum. *Oceanol. Acta* **14**, 311–324 (1991).

103. S. de Villiers, M. Greaves, H. Elderfield, An intensity ratio calibration method for the accurate determination of Mg/Ca and Sr/Ca of marine carbonates by ICP-AES. *Geochem. Geophys. Geosyst.* **3**, 1001 (2002).

104. P. Anand, H. Elderfield, M. H. Conte, Calibration of Mg/Ca thermometry in planktonic foraminifera from a sediment trap time series. *Paleoceanography* **18**, 1050 (2003).

105. T. Tyrrell, R. E. Zeebe, History of carbonate ion concentration over the last 100 million years. *Geochim. Cosmochim. Acta* **68**, 3521–3530 (2004).

106. E. Anagnostou, E. H. John, K. M. Edgar, G. L. Foster, A. Ridgwell, G. N. Inglis, R. D. Pancost, D. J. Lunt, P. N. Pearson, Changing atmospheric CO2 concentration was the primary driver of early Cenozoic climate. *Nature* **533**, 380–384 (2016).

107. J. Horita, H. Zimmermann, H. D. Holland, Chemical evolution of seawater during the Phanerozoic: Implications from the record of marine evaporites. *Geochim. Cosmochim. Acta* **66**, 3733–3756 (2002).

108. R. M. Coggon, D. A. H. Teagle, C. E. Smith-Duque, J. C. Alt, M. J. Cooper, Reconstructing past seawater Mg/Ca and Sr/Ca from mid-ocean ridge flank calcium carbonate veins. *Science* **327**, 1114–1117 (2010).

109. D. Evans, N. Sagoo, W. Renema, L. J. Cotton, W. Müller, J. A. Todd, P. K. Saraswati, P. Stassen, M. Ziegler, P. N. Pearson, P. J. Valdes, H. P. Affek, Eocene greenhouse climate revealed by coupled clumped isotope-Mg/Ca thermometry. *Proc. Natl. Acad. Sci.* **115**, 1174–1179 (2018).

110. F. J. Hasiuk, K. C. Lohmann, Application of calcite Mg partitioning functions to the reconstruction of paleocean Mg/Ca. *Geochim. Cosmochim. Acta* **74**, 6751–6763 (2010).

111. T. Dunkley Jones, D. J. Lunt, D. N. Schmidt, A. Ridgwell, A. Sluijs, P. J. Valdes, M. Maslin, Climate model and proxy data constraints on ocean warming across the Paleocene–Eocene Thermal Maximum. *Earth-Science Rev.* **125**, 123–145 (2013).

112. D. Evans, B. S. Wade, M. Henehan, J. Erez, W. Müller, Revisiting carbonate chemistry controls on planktic foraminifera Mg / Ca: Implications for sea surface temperature and hydrology shifts over the Paleocene-Eocene Thermal Maximum and Eocene-Oligocene transition. *Clim. Past.* **12**, 819–835 (2016).

113. D. Evans, C. Brierley, M. E. Raymo, J. Erez, W. Müller, Planktic foraminifera shell chemistry response to seawater chemistry: Pliocene-Pleistocene seawater Mg/Ca, temperature and sea level change. *Earth Planet. Sci. Lett.* **438**, 139–148 (2016).

114. E. C. Hopmans, S. Schouten, J. S. Sinninghe Damsté, The effect of improved chromatography on GDGT-based palaeoproxies. *Org. Geochem.* **93**, 1–6 (2016).

115. E. C. Hopmans, J. W. H. Weijers, E. Schefuß, L. Herfort, J. S. Sinninghe Damsté, S. Schouten, A novel proxy for terrestrial organic matter in sediments based on branched and isoprenoid tetraether lipids. *Earth Planet. Sci. Lett.* **224**, 107–116 (2004).

116. S. Schouten, E. C. Hopmans, E. Schefuß, J. S. Sinninghe Damsté, Distributional variations in marine crenarchaeotal membrane lipids: A new tool for reconstructing ancient sea water temperatures? *Earth Planet. Sci. Lett.* **204**, 265–274 (2002).

117. Y. G. Zhang, C. L. Zhang, X. L. Liu, L. Li, K. U. Hinrichs, J. E. Noakes, Methane Index: A tetraether archaeal lipid biomarker indicator for detecting the instability of marine gas hydrates. *Earth Planet. Sci. Lett.* **307**, 525–534 (2011).

118. C. I. Blaga, G. J. Reichart, O. Heiri, J. S. Sinninghe Damsté, Tetraether membrane lipid distributions in water-column particulate matter and sediments: A study of 47 European lakes along a north-south transect. *J. Paleolimnol.* **41**, 523–540 (2009).

119. J. W. H. Weijers, K. L. H. Lim, A. Aquilina, J. S. S. Damsté, R. D. Pancost, Biogeochemical controls on glycerol dialkyl glycerol tetraether lipid distributions in sediments characterized by diffusive methane flux. *Geochem. Geophys. Geosyst.* **12**, Q10010 (2011).

120. K. W. R. Taylor, M. Huber, C. J. Hollis, M. T. Hernandez-Sanchez, R. D. Pancost, Re-evaluating modern and Palaeogene GDGT distributions: Implications for SST reconstructions. *Glob. Planet. Change* **108**, 158–174 (2013).

121. C. L. O’Brien, S. A. Robinson, R. D. Pancost, J. S. Sinninghe Damsté, S. Schouten, D. J. Lunt, H. Alsenz, A. Bornemann, C. Bottini, S. C. Brassell, A. Farnsworth, A. Forster, B. T.

Huber, G. N. Inglis, H. C. Jenkyns, C. Linnert, K. Littler, P. Markwick, A. McAnena, J. Mutterlose, B. D. A. Naafs, W. Püttmann, A. Sluijs, N. A. G. M. van Helmond, J. Vellekoop, T. Wagner, N. E. Wrobel, Cretaceous sea-surface temperature evolution: Constraints from TEX_{86} and planktonic foraminiferal oxygen isotopes. *Earth-Sci. Rev.* **172**, 224–247 (2017).

122. Y. G. Zhang, M. Pagani, Z. Wang, Ring index: A new strategy to evaluate the integrity of TEX_{86} paleothermometry. *Paleoceanography* **31**, 220–232 (2016).

123. J. S. Sinninghe Damsté, Spatial heterogeneity of sources of branched tetraethers in shelf systems: The geochemistry of tetraethers in the Berau River delta (Kalimantan, Indonesia). *Geochim. Cosmochim. Acta* **186**, 13–31 (2016).

124. R. D. Müller, J. Cannon, X. Qin, R. J. Watson, M. Gurnis, S. Williams, T. Pfaffelmoser, M. Seton, S. H. J. Russell, S. Zahirovic, GPlates: Building a virtual earth through deep time. *Geochem. Geophys. Geosyst.* **19**, 2243 –2261 (2018).

125. K. J. Matthews, K. T. Maloney, S. Zahirovic, S. E. Williams, M. Seton, R. D. Müller, Global plate boundary evolution and kinematics since the late Paleozoic. *Glob. Planet. Change.* **146**, 226–250 (2016).

126. R. Schlitzer, Data analysis and visualization with ocean data view. *C. Bull. SCMO* **43**, 9–13 (2015).
